# Light-Driven Intraoctahedral Halide Isomerization in Two-Dimensional Mixed Halide Perovskites

**DOI:** 10.1021/jacs.5c15542

**Published:** 2026-01-13

**Authors:** Wenxin Mao, Enamul Haque, Stephanie A. Bird, Milos Dubajic, Yang Lu, Xian Wei Chua, Zhou Xu, Xinjuan Li, Mitko Oldfield, Jialu Li, Wenqi Yan, Christopher R. Hall, Qingdong Lin, Jie Zhao, Anthony S. R. Chesman, Gary Beane, Agustin Schiffrin, Caterina Ducati, Nikhil Medhekar, Samuel D. Stranks, Udo Bach

**Affiliations:** † Department of Chemical Engineering and Biotechnology, 2152 University of Cambridge, Philippa Fawcett Drive, Cambridge CB3 0AS, U.K.; ‡ ARC Centre of Excellence in Exciton Science, Department of Chemical and Biological Engineering, 2541 Monash University, Clayton, Vic 3800, Australia; § ARC Centre of Excellence in Future Low-Energy Electronics Technologies, Department of Materials and Science Engineering, 2541 Monash University, Clayton, Vic 3800, Australia; ∥ 5419 Australian Synchrotron, Clayton, Vic 3168, Australia; ⊥ Monash Centre for Electron Microscopy, 2541 Monash University, Clayton, Vic 3800, Australia; # Department of Materials Science and Metallurgy, 2152 University of Cambridge, Cambridge CB3 0FS, U.K.; ∇ ARC Centre of Excellence in Future Low-Energy Electronics Technologies, School of Physics and Astronomy, 2541 Monash University, Clayton, Vic 3800, Australia; ○ ARC Centre of Excellence in Exciton Science, The University of Melbourne, Melbourne, Vic 3010, Australia; ◆ 2221 CSIRO Manufacturing, Clayton, Vic 3168, Australia

## Abstract

Two-dimensional metal halide perovskites are emerging materials for quantum light emission and neuromorphic computing owing to their quantum-confined structures and tunable optoelectronic properties. Beyond structural dimensionality, the presence of multiple crystallographically distinct halide sites within a single metal halide octahedron presents a unique opportunity to engineer functionality at the subunit-cell level. Here, we report a light-driven, reversible halide-ion isomerization in single-crystalline BA_2_PbBr_*x*_I_4–*x*_ (BA = butylammonium, *x* = 1–3), where ions switch between distinct local configurations within individual PbX_6_^4–^ octahedra, without long-range migration or macroscopic phase segregation. Through a combination of hyperspectral imaging, in situ X-ray diffraction, and first-principles calculations, we demonstrate that this intraoctahedral halide site switching modulates the optical bandgap by ∼0.1 eV and enables an estimated reversible electronic bandgap shift of up to ∼0.5 eV. Density functional theory reveals that these changes stem from a redistribution of valence band character, effectively creating chemically distinct optoelectronic isomers that can be activated by light. These results uncover a mechanism of structurally encoded, site-selective photoisomerization in 2D perovskites, offering a new strategy for reconfigurable optoelectronic devices, nonvolatile optical memory, and quantum photonics.

## Graphical abstract

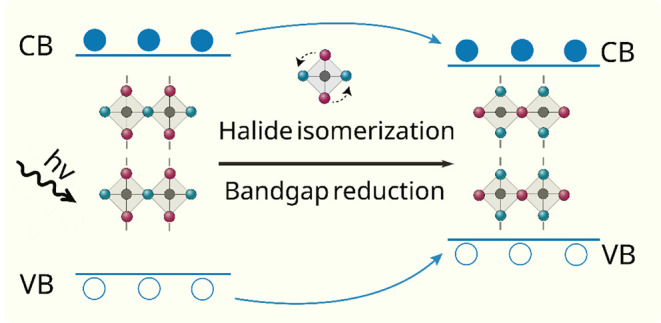

## Introduction

Two-dimensional lead halide perovskites (2DHPs) have emerged as a promising class of materials for next-generation optoelectronic devices, evolving from photovoltaic absorbers^1–3^ and LED emitters^4,5^ into a broadly relevant platform for nanophotonics,^6,7^ spintronics,^8,9^ and even ferroelectric devices.^10^ These materials possess a naturally layered structure comprising alternating inorganic PbX_6_^4–^ sheets and organic spacer layers, resulting in a quantum-well architecture with strong dielectric and quantum confinement effects.^11^ Similar to their three-dimensional (3D) lead halide perovskite counterparts, mixed halide chemistry can also be applied to 2D perovskites, enabling wide bandgap tuning while potentially suppressing ion migration. Unlike 3DMHPs where photoinduced halide segregation has been broadly reported in both single crystals and polycrystalline films, 2D mixed halide perovskites (2DMHPs) have shown suppressed segregation behavior attributed to their reduced ion migration and strong exciton-lattice interactions.^12^ Nonetheless, the fundamental nature of segregation in 2DMHPs remains under debate. Importantly, previous photoinduced investigations in 2DMHPs have been limited to polycrystalline thin films, where there may be additional contributions from defects, initial inhomogeneous halide distributions, and interfacial effects.^13^ In contrast, photoluminescence (PL) microscopy studies using single-crystal 2DMHPs have yet to observe any characteristic low bandgap segregated domains typically seen in 3D perovskites under illumination, suggesting that photoinduced halide segregation (PHS) may not be an intrinsic property of these materials.^14,15^

Distinct from 3DMHPs where all halide sites are corner-shared through the PbX_6_^4–^ octahedra networks, an inherent structural feature of 2DHPs is the presence of two distinct halide lattice sites: terminal axial sites (T-site) and bridging equatorial sites (B-site). Toso et al. recently reported that iodide ions (I^–^) tend to occupy T-site while bromide ions (Br^–^) prefer B-site to form a stacked ordering structure, which is contrary to the common assumption that Br^–^ and I^–^ are homogeneously distributed in the lattice.^16^ Hope et al. provided further evidence using 2D isotropic–anisotropic correlation ^207^Pb NMR and relativistic density functional theory (DFT) calculations to support such unique halide occupancy preference in 2DMHPs with butylammonium (BA) as a spacer.^17^ Despite this observation, the influence of such site-selective halide stacking on light-induced structural dynamics, stability, and optoelectronic properties remains unexplored.

Here, we investigate single crystals of the 2D mixed halide perovskite series BA_2_PbBr_*x*_I_4–*x*_ (*x* = 1, 2, 3) and show that they exhibit a unique halide stacking preference under thermal equilibrium, with Br^–^ and I^–^ showing a preference for occupying the B- and T-sites of the PbX_6_^4–^ octahedra, respectively. Hyperspectral photoluminescence and absorptance imaging reveal the emergence of a new homogeneous phase upon above-bandgap photoexcitation. This phase originates from a halide site-specific photoisomerization, in which T-site I^–^ and B-site Br^–^ exchange positions locally within PbX_6_^4–^ octahedra without long-range halide migrations. In situ temperature-dependent X-ray diffraction confirms that this exchange is accompanied by lattice reconfiguration—an in-plane expansion with simultaneous interlayer contraction. These photoactivated and dark-relaxed states represent chemically distinct isomers with different physical properties. Density functional theory (DFT) calculations predict a ∼0.5 eV bandgap narrowing in the photoswitched structure, driven by a shift in valence-band edge contributions from T-site to B-site iodide. Importantly, the process proceeds without macroscopic phase segregation, preserving structural uniformity. Composition-dependent studies further show that iodide-rich crystals exhibit lower photoactivation thresholds, consistent with the greater polarizability and lattice softness of iodide. Together, these results establish a light-driven, reversible halide isomerization pathway for bandgap control, opening opportunities for quantum photonics, reconfigurable nanophotonics, and nonvolatile optical memory.

## Microscopic Imaging of Photoinduced Phase Transextension for 2DMHP Single Crystals

High-quality crystals were synthesized using a space-confining method (Method), yielding lateral sizes ranging from 0.01 to 4 mm^2^ and controlled thicknesses of 300–500 nm.^18^ The layered nature of the crystals is evident from atomic force microscopy (AFM), with monolayer step heights of ∼1.3 nm (Figure S1). EDX measurements of BA_2_PbBr_2_I_2_ single crystals reveal uniform distributions of both Br and I across lateral spans greater than 200 μm and throughout the entire vertical cross-section of the crystals, confirming the absence of any preexisting segregated phases (Figures S2 and S3). The BA_2_PbBr_2_I_2_ crystal structure was verified by single-crystal X-ray diffraction (SCXRD) at 100 K, which revealed a high iodide occupancy of ∼68% at the T-site—approximately twice that at the B-site, with bromide showing the opposite site preference (Figures 1a and S4–S5). This is consistent with recent reports of well-ordered layered mixed halide perovskites with different halide occupancy preferences.^16,17^ Such a unique halide arrangement forms a distinct difference with the random halide distribution in 3DMHPs (Figure 1e).^19^

**Figure 1:**
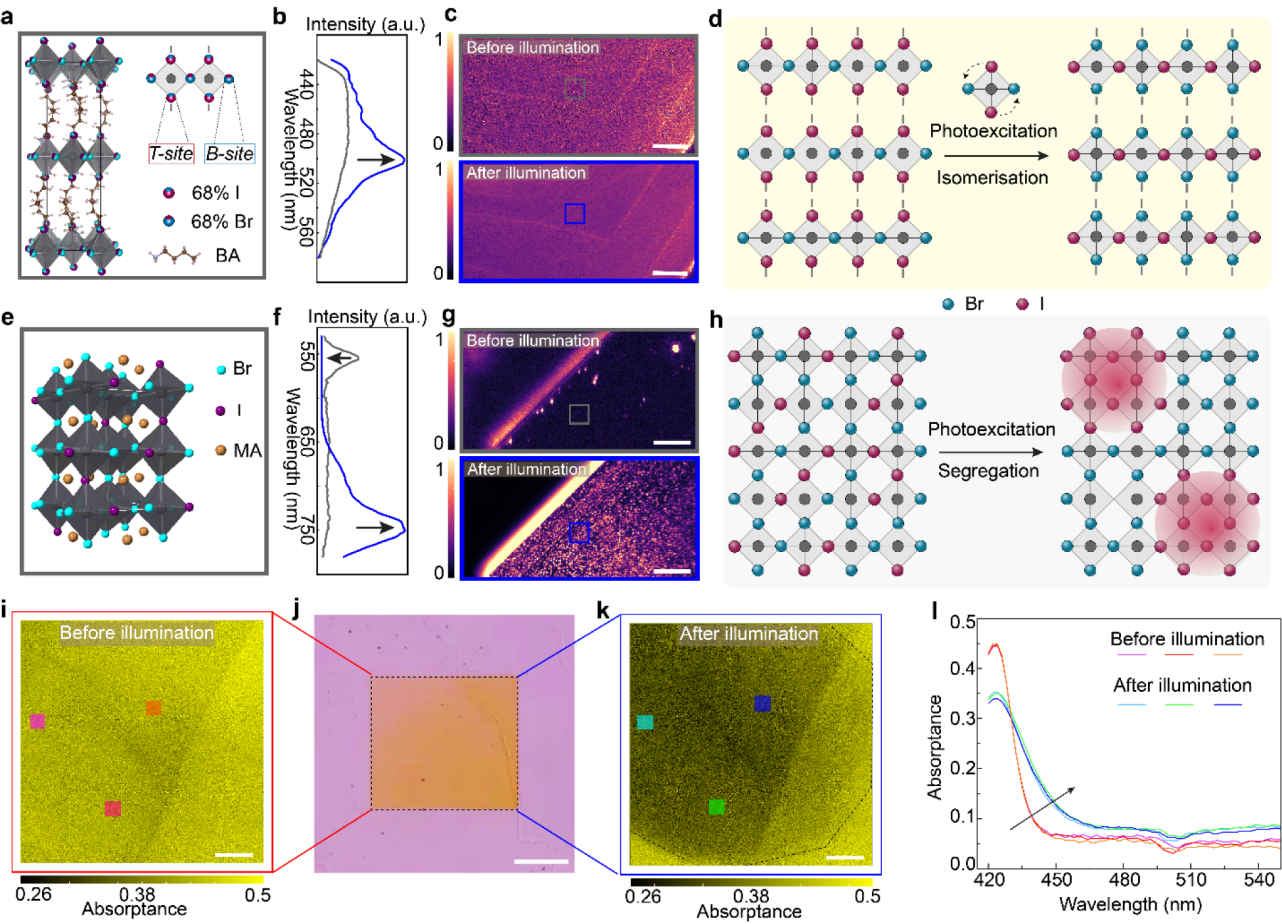
Microscopic structure–property correlation of of layered BA_2_PbBr_2_I_2_ thin crystals. (a) Crystal structure of 2D BA_2_PbBr_2_I_2_ from SCXRD data measured at 100 K and corresponding schematics showing 68% Br at B-site and 68% I at T-site. (b) Single PL spectra for BA_2_PbBr_2_I_2_ without (gray curve) and with photoexcitation (blue curve). (c) Hyperspectral PL maps of a thin BA_2_PbBr_2_I_2_ single crystal. The top and bottom images represent before and after photoexcitation by a 405 nm CW laser for 5 min. The central wavelength of the PL maps is 500 nm, which is the iodide-rich phase wavelength window. (d) Schematic illustration of photoisomerization for 2D BA_2_PbBr_2_I_2_. Note: in this diagram, all I atoms are positioned at T-site for demonstrating the halide switching purpose. (e) Schematic illustration of the random halide distribution in a 3D MAPbBr_*x*_I_3–*x*_ mixed halide perovskite network. (f) Single PL spectra for MAPbBr_*x*_I_3–*x*_ without (gray curve) and with photoexcitation (blue curve). (g) Hyperspectral PL maps of a thin MAPbBr_*x*_I_3–*x*_ single crystal. The top and bottom images represent before and after photoexcitation by a 405 nm CW laser with an excitation intensity of 80 mW/cm^2^ for 5 min. The central wavelength of the PL maps is 750 nm, which is the iodide-rich phase wavelength window. (h) Schematic illustration of light-induced halide segregation for 3D MAPbBr_*x*_I_3–*x*_. (i, k) Absorptance maps measured on a thin BA_2_PbBr_2_I_2_ crystal before (i) and after excitation (k). The maps in (i) and (k) are taken at the central wavelength of 424 nm. The photoexcitation region is shown in the dashed area. (j) Optical image presenting a thin BA_2_PbBr_2_I_2_ crystal where the central squared region is the photoexcited region. (l) Absorptance spectra before and after photoexcitation at three isolated points labeled in (i) and (k). The scale bars in all microscopic images are 20 μm.

To probe the influence of photoexcitation on the ordered structure, we investigated the PL response of thin mixed-halide single crystals under pre- and postphotoexcited conditions using hyperspectral microscopy, enabling spatially resolved analysis of photoinduced phase transitions. We first investigated the halide-ordered 2D BA_2_PbBr_2_I_2_ thin single crystals. Prior to the extended 5 min photoexcitation, it shows a broad PL emission spanning 420–600 nm (gray curve in Figure 1b), which can be attributed to self-trapped exciton (STE) recombination.^12,20,21^ To confirm that the observed PL broadening arises from intrinsic bulk properties rather than surface-related trap states, cathodoluminescence (CL) using high-energy primary electrons with a deeper excitation depth (Figure S6) reveals similar bandwidth photoluminescence, confirming that the spectral broadening originates from bulk structural features rather than surface defects (Figure S7). Such broadband emission is uniform across the entire illuminated crystal (Figure 1c top panel) without phase segregation or iodide-rich domains (Figure S8). As a reference, we also examined a 3D MAPb­(Br_*x*_I_1–*x*_)_3_ MHP single crystal with an estimated Br:I ratio of around 4:1.^14^ As shown in Figure 1f and g, after exposed to a 405 nm continuous wave (CW) laser with an intensity of 80 mW/cm^2^ for 5 min, the PL map at 750 nm reveals pronounced phase segregation with isolated low bandgap domains forming and extending across the crystal (Figure S9). This segregation is accompanied by a suppression of Br-rich emission, which can be attributed to the trapping of carriers into the lower-bandgap I-rich domains (Figure 1f).

For the 2D structure with photoexcitation by the same 405 nm CW laser at an intensity of 80 mW/cm^2^ for 5 min, a following hyperspectral PL mapping (Figure 1c bottom panel and Figure S10) reveals the same homogeneity with an emergence of a new dominant emissive phase centered at 500 nm (blue curve in Figure 1b). We observe that the formation of this new phase tends to begin at crystal edges and interlayers due to the weak bonding environment and higher atomic vacancies at the layer terminals,^22,23^ accelerating the phase transition (Figure S11). This new phase can be further distributed homogeneously across the entire excitation area without macroscopic halide segregation (Figures S12–S13). The distinct photoinduced halide rearrangements in 2D mixed halide perovskite BA_2_PbBr_2_I_2_ point to a localized process (Figure 1d), rather than the halide segregation presented in 3DMHPs (Figure 1h). A mechanistic comparison of photoinduced halide redistribution between 3DMHP and 2DMHP is shown in Figure S14.

To further confirm the homogeneity of this photoinduced new phase, spatially resolved absorptance maps were measured before and after photoexcitation by a 405 nm CW laser (Figure 1i–k). The uniform spectral shift across three representative points within the photoexcited region (Figure 1l) confirms the homogeneity of the new phase. A clear red-shift in the absorption onset corresponds to a reduction in optical bandgap by approximately 0.1 eV, indicating that the phase transition directly alters the photophysical properties of the material.

## Structural Analysis of Photoisomerization

We now move on to the discussion of how such photoinduced phase transition links to their unique halide occupancies by exploring their structural changes under photoexcitation. We note that all single-crystal XRD (SCXRD) results were measured at a Synchrotron SCXRD facility with an ultrafast Eiger 16 M detector that allows a fast 36 s data collection for a complete set of high-resolution diffraction patterns. Figure 2a shows the T-site iodine occupancy ratios for BA_2_PbBr_2_I_2_ extracted from temperature-dependent SCXRD data measured either in ambient light or after photoexcitation. At 100 and 200 K, the T-site I^–^ occupancy remains unaltered with no measurable halide redistribution over a period of 80 min photoexcitation by a 405 nm LED with an intensity of ∼50 mW/cm^2^ (Figure 2a and Table S1). In a crystal measured at 293 K, ∼60% of I^–^ remains at the T-site. The slight difference at 293 K compared to 100 K indicates that at this temperature, thermal energy allows higher mobility of halide ions. Further photoexcitation for 20 and 40 min under the same conditions induces clear halide redistribution, with approximately 36% of I^–^ remaining at T-sites, corresponding to ∼41% of halides switching their positions relative to the structure in the dark state (Figure 2a and Tables S2 and S3). This partial exchange gives rise to subtle but distinct structural distortions, most evident in the in-plane Pb–X–Pb (X = Br, I) bond angles. The in-plane structural changes extracted from the SCXRD data are quantified in Figure 2b. At 293 K, the Pb–X–Pb angle increases by 1.3°, whereas the Pb–Pb distance decreases by only 0.017 Å after 20 min of photoexcitation (Figure 2b). Mechanistically, the substitution of bromine by the larger iodine within the PbX_6_^4–^ octahedra leads to an increase in the local Pb–X bond lengths, which in turn pushes neighboring Pb atoms further apart and relaxes the bond angles. The negligible change in the Pb–Pb distance reflects the fact that partial halide exchange only modestly perturbs the average Pb–X bond length when assuming Br and I share the same crystallographic site. No further evolution of either the Pb–Br–Pb angles or the Pb–Pb distances is observed upon extending the photoexcitation to 40 min, indicating the absence of major symmetry changes, such as phase segregation induced by halide switching. Full SCXRD data are shown in Table S2.

**Figure 2:**
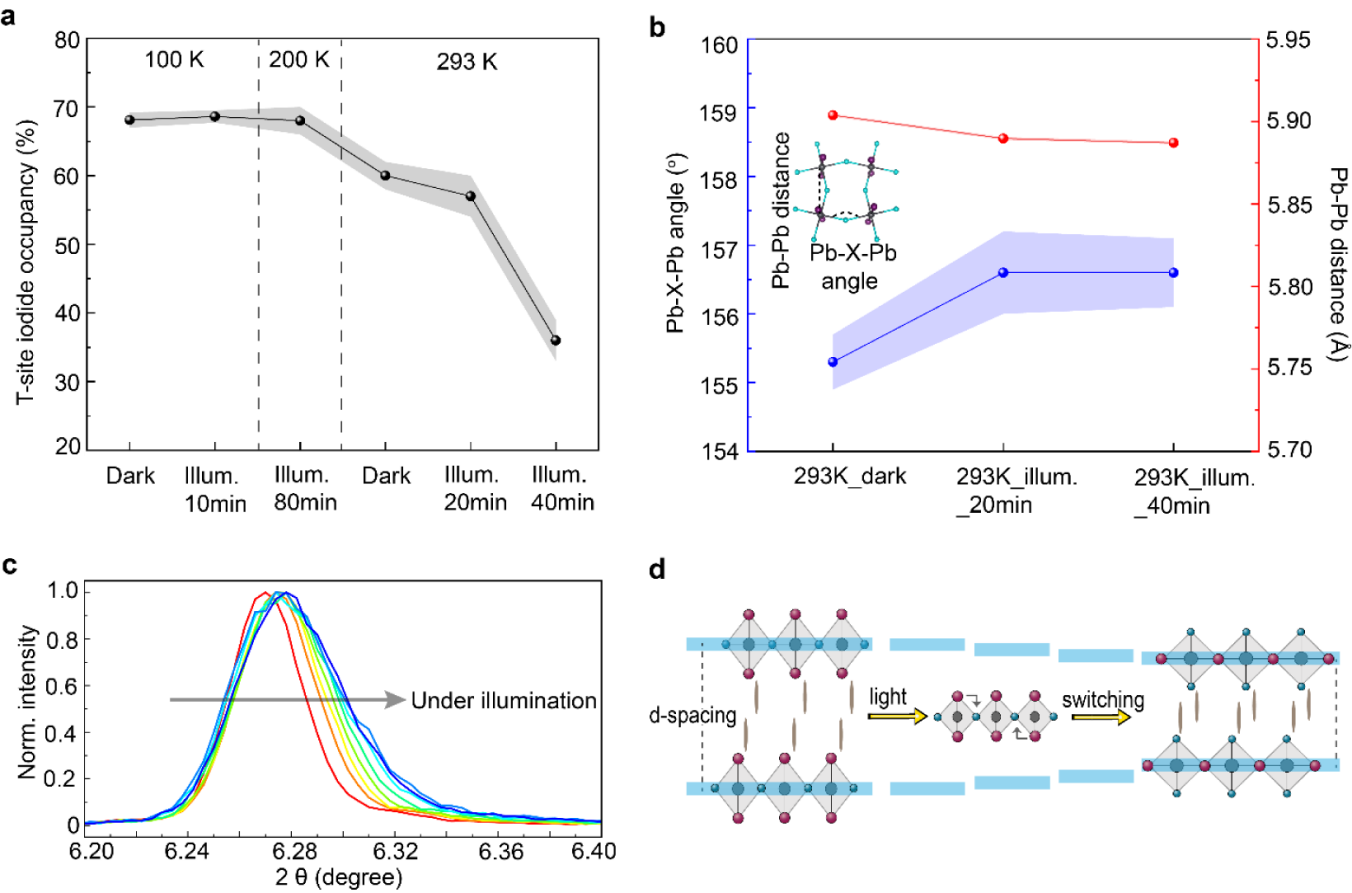
Crystal structure characterizations of light-induced ion switching for BA_2_PbBr_2_I_2_. (a) T-site iodine occupancy ratios derived from temperature-dependent SCXRD measurements of BA_2_PbBr_2_I_2_ at 100, 200, and 293 K under dark and photoexcited conditions. A 405 nm blue LED with an intensity of ∼50 mW/cm^2^ was used as excitation. Full SCXRD data are available in Supplementary Tables S1–S3. (b) Analysis of changes in the average equatorial Pb–X–Pb angle (X = Br/I) and Pb–Pb distance for the structures measured at 293 K before and after photoexcitation by the above LED source. Inset: schematic illustration of Pb–Pb distance and Pb–X–Pb angle from a top view. (c) Room-temperature XRD of thin BA_2_PbBr_2_I_2_ crystals on a glass substrate to analyze out-of-plane changes under photoexcitation. The crystals were illuminated for 60 s before each XRD scan. Each measurement scanned from 5.522° to 7.018° and took 3 min to complete. Excitation: a 385 nm LED with intensity around 100 mW/cm^2^. (d) Schematic illustration of the photoinduced halide switching mechanism resulting in *d*-spacing reduction, which can be calculated following Bragg’s Law: *n*λ = 2*d*sinθ.

We next analyze the changes in out-of-plane structures due to photoinduced halide switching. Figure 2c presents the high-resolution XRD result for thin BA_2_PbBr_2_I_2_ crystals under a 385 nm LED illumination measured at room temperature. Upon photoexcitation, the (002) diffraction peak shifts from 6.27° to 6.28°, corresponding to a reduction in the interlayer spacing (*d*-spacing) from 14.09 to 14.06 Å, which can be calculated following the Bragg’s Law. This contraction is consistent with halide switching, where longer Pb–I bonds at T-sites are replaced by shorter Pb–Br bonds. In addition, the expanded lateral space due to the longer Pb–I bonds at bridging sites allows the BA molecules to occupy less space for the vertical stacking to maintain a stable structure, thus further reducing the interlayer distance. A schematic illustration of this *d*-spacing contraction due to ion switching is presented in Figure 2d. No new XRD peaks or peak splitting emerges during illumination, which supports that the halide exchange occurs homogeneously across the crystal without generating separate Br-rich or I-rich domains, consistent with the photoluminescence results shown earlier. In other words, the photoexcited lattice remains a single phase with its original average symmetry intact, while only altering the halide site occupancies. A noticeable diffraction peak broadening (Figure 2c) together with an intensity drop (Figure S15) during illumination suggests that while the structure stays globally coherent, the photoinduced halide motion introduces lattice strain or local disorder. Notably, the illumination-induced temperature increase does not play a major role for this halide switching process. The SCXRD temperature was precisely regulated using a nitrogen cryojet, whereas the room-temperature measurements showed only a 0.5 K increase, indicating that illumination-induced heating is not the primary driving force for halide switching (Figure S16). In addition, this photoisomerization is reversible upon removal of the external photoexcitation, as demonstrated by both structural and optical analyses (Figures S15 and S17). We note that a complete dark recovery will take over 9 h (Figure S18). To assess reversibility, we exposed the thin crystal to multiple light-on (300 s) and light-off cycles until the PL peak reduced to 50 ± 10% of its maximum. Figure S19 shows time-dependent PL results for three cycles. During the first 300 s illumination, PL rises from a low broad peak, consistent with the unswitched phase. Recovery is also slow with ∼60% intensity remaining after 110 min in the dark. In subsequent cycles, both switching and recovery dynamics accelerate: with the same 300 s illumination, the PL intensity increases by ∼72% and ∼172%, while after 60 min in the dark, only ∼50% and ∼40% persist for cycles 2 and 3, respectively. This can be attributed to the partially recovered states that lower the barriers for both switching and recovery. These results reveal a photoactivated yet thermally gated photoisomerization process: below the thermal threshold (between 200 and 293 K), the halide sublattice is photoinactive within a measurable time frame, but once that threshold is exceeded, light absorption drives a collective, uniform halide rearrangement that subtly modifies the lattice spacing and geometry without triggering phase separation.

## Simulation of Halide Switching

We model the photoinduced halide switching pathway by calculating the minimum energy path (MEP). To clearly visualize the energy diagrams during halide switching, we use a simplified structural model with all I^–^ at the T-site to a fully switched phase with all I^–^ at the B-site. Starting from the initially unperturbed (before photoexcitation) phase *R*_0_ as the ground state, under photoexcitation, the system evolves through sequential intermediate configurations *R*_1_, *R*_2_, ..., *R_N_* along the photoinduced switching pathway. Each configuration *R*_*i*_ represents a snapshot of the atomic coordinates during this transition. For each intermediate configuration, the total energy is calculated as *E*_*i*_ = *E*(*R*_*i*_). The activation energy *E*_a_ for the simultaneous switching of both Br and I ions is then estimated as the maximum energy difference along the minimum energy path:

$$E_{a}=\max\left\{E\left(R_{i}\right)-E\left(R_{0}\right)\right\},\;i=1,\;2,\;...,\;N-1$$

Where *E*_0_ = *E*(*R*_0_) is the ground state energy of the original phase. Based on the fully optimized atomic configurations of the initial phase (*R*_0_) and the final photoswitched state (*R_N_*), we constructed a series of 10 intermediate configurations (*R*_1_, *R*_2_, ..., *R_N_*_–1_), representing partially switched structures along the transition pathway. These intermediate images were then simultaneously optimized along the reaction coordinate using the nudged elastic band (NEB) method to locate the transition state (saddle point) and the MEP of the switching process. The NEB algorithm finds the minimum energy path between known end points by adding artificial spring forces between adjacent structures and projecting out the component of the true force that is parallel to the path, so that only the perpendicular component of the potential energy gradient drives each structure toward a local minimum. Details of mathematical calculations are shown in the Methods section.

The resulting energy profile presented in Figure 3a shows a single peak: the activation barrier for simultaneous switching of all 16 halide ions is about 0.76 eV relative to that of the original phase. Because this barrier is moderate, partial switching where only some ions can occur at lower energy. Indeed, the partially switched intermediates along the NEB path represent metastable states that could be thermally or photoactivated below the full 0.76 eV threshold. The NEB results reveal that ion switching requires overcoming a single concerted barrier while partially switched intermediates along the NEB path representing metastable states could be thermally or photoactivated below the full 0.76 eV threshold. A diagram of this ion switching pathway is shown in Figure 3b.

**Figure 3:**
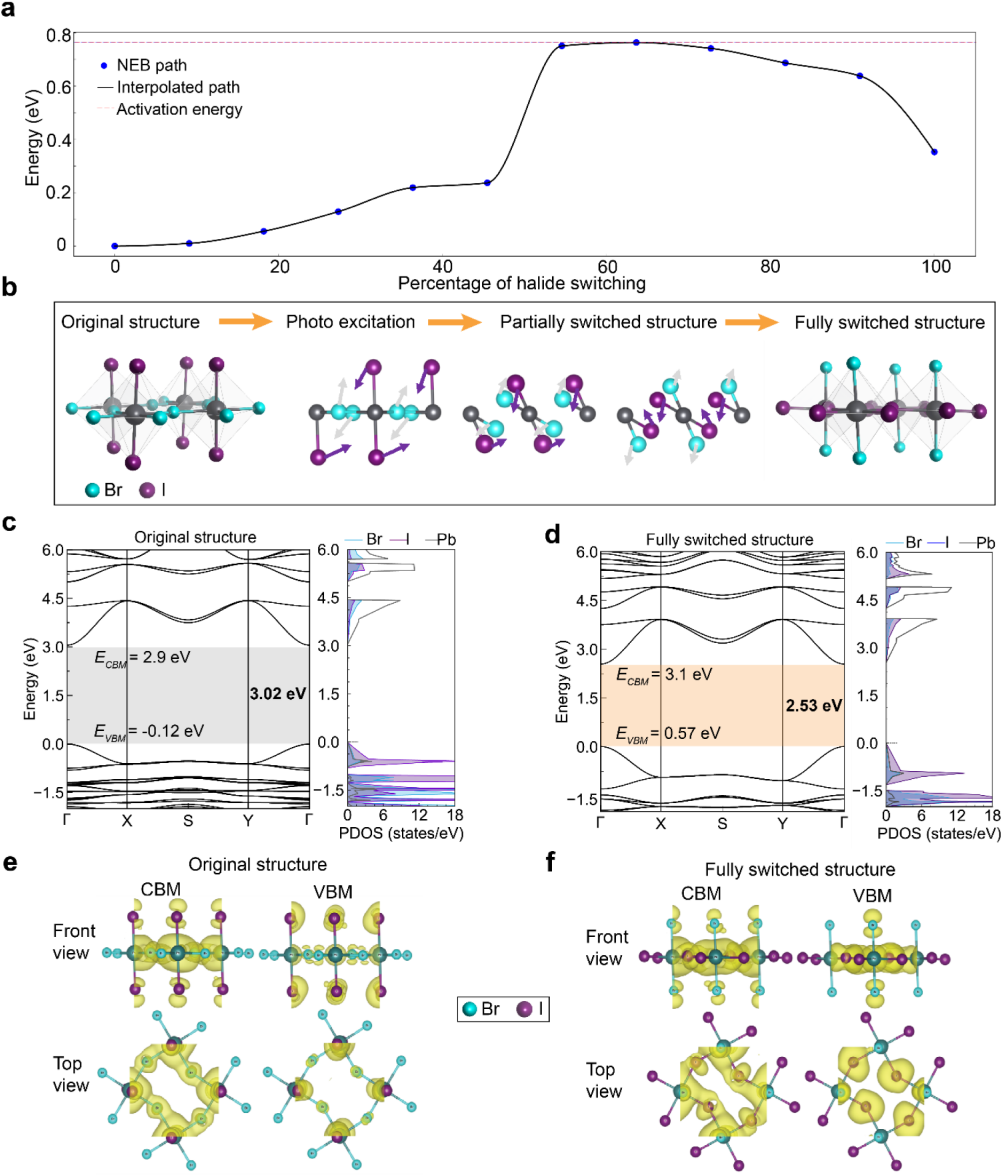
DFT calculation of the activation energy for halide switching for BA_2_PbBr_2_I_2_. (a) Activation energy diagram from the original structure to the switched structure. The energy diagram plotted in this figure represents the average energy. (b) Schematic of halide switching steps under photoexcitation. (c, d) Calculated electronic band structures and projected density of states (PDOS) of (c) the original structure and (d) the halide switched structure. (e, f) Corresponding band-decomposed charge density distribution of the conduction band minima (CBM) and valence band maxima (VBM) for (e) the original structure and (f) the halide switched structure.

To investigate the electronic origins of the observed optical bandgap reduction (Figure 1l), we calculate the band structures of both the original and fully switched phases using the many-body GW approximation with spin–orbit coupling effect (GW+SOC) (Method). Both the original and fully switched phases are found to be direct bandgaps. The calculated bandgaps shrink from about 3.0 eV in the initial (all-T-site) structure to 2.5 eV in the final (all-B-site) structure (Figure 3c) and 2.5 eV for the switched structure (Figure 3d). Major change occurs in the valence band. In the initial structure, the VBM consists mainly of antibonding combinations of I 5p and Pb 6s orbitals, whereas the CBM is Pb 6p. The bandgap narrowing in the switched phase is primarily attributed to a pronounced upward shift in the valence band maximum (VBM). When I^–^ ions switch from T-sites to B-sites, the I–Pb bonding environment strengthens, where B-site I^–^ bonds to two Pb^2+^ rather than a single Pb^2+^ due to the enhanced hybridization of I 5p with Pb 6s. The VBM increases its energy, thus reducing the bandgap. Our calculated charge density analysis further confirms this effect. As shown in Figure 3e and f, the valence charge clouds on I and neighboring Pb significantly interpenetrate in the switched phase (with all I^–^ on bridging sites) indicating stronger I–Pb interactions, which corroborates the upward shift of the VBM. This matches the predicted increase in I 5p character at the VBM and the observed 0.5 eV bandgap narrowing. Our combined NEB and band-structure analysis thus provides a coherent theoretical picture: the photoinduced ionic motion changes the lattice geometry, which in turn tunes the orbital hybridization and optical properties in a consistent way.

## Impact of Halide Ratios on Photoisomerization

The impact of halide composition on the photoisomerization in 2DHPs was systematically investigated using single crystals of three representative compositions: BA_2_PbBr_3_I, BA_2_PbBr_2_I_2_, and BA_2_PbBrI_3_. All three structures share a similar halide distribution in their ground states, with a large fraction of I^–^ preferentially occupying T-sites and Br^–^ populating the B-sites (Figure 4a,d,g). Full lattice constants for these structures are provided in Tables S4 and S5.

**Figure 4:**
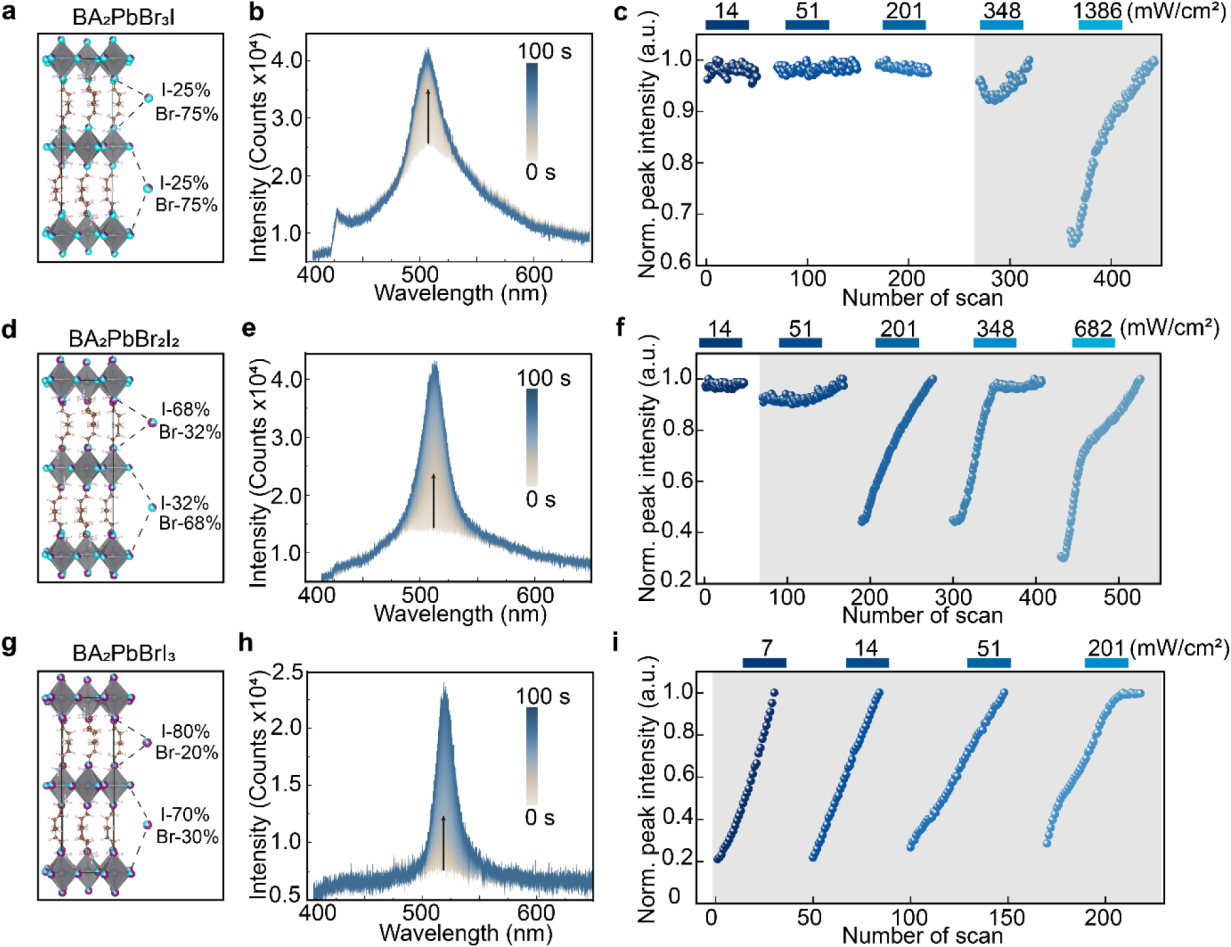
Photoisomerization of 2DMHPs with different halide ratios. (a, d, g) Crystal structures of BA_2_PbBr_3_I (a), BA_2_PbBr_2_I_2_ (d), BA_2_PbBrI_3_ (g). The purple and blue spheres represent I and Br atoms, respectively. The shared spheres represent occupancy ratios of I and Br atoms taking those sites. (b-h) Time-dependent PL spectra at above photoisomerization excitation threshold for the three different halide ratios. The spectra are taken continuously for 100 s. (c-i) PL peak intensity trace tracking at different excitation intensities for the above three compositions. The shaded area indicates the turn-on excitation threshold of photoisomerization for the three halide ratios.

To assess the influence of different Br/I ratios on the photoisomerization thresholds of these materials, we monitored the evolution of the stabilized PL peak intensity as a function of excitation intensity. The onset of photoisomerization was identified by the emergence and subsequent dominance of an emission peak around 500–510 nm when the photoexcitation density is above the photoisomerization threshold (Figure 4b,e,h). In Br_3_I (Figure 4c), the PL intensity remained unchanged at excitation intensities until 348 mW/cm^2^, where a subtle decline followed by a modest increase in PL peak intensity at 500 nm indicates partial halide switching. A more pronounced increase in PL intensity is observed at 1386 mW/cm^2^, confirming the occurrence of substantial halide redistribution. In contrast, Br_2_I_2_ and BrI_3_ exhibit photoisomerization thresholds at significantly lower excitation intensities of 51 mW/cm^2^ and 7 mW/cm^2^ (lowest intensity of the light source), respectively, corresponding to reductions by factors of around 7 and 50 relative to Br_3_I (Figure 4f and i). These findings suggest a strong dependence of the photoactivation barrier on the halide composition. The markedly lower threshold in the iodide-rich BrI_3_ implies a reduced energy barrier for halide switching, which is likely due to the increased polarizability and greater lattice softness of I^–^ compared to Br^–^. A full result of excitation intensity-dependent stabilized PL spectra for all the compositions is presented in Figures S20-S22. Unlike the halide segregation commonly observed in 3DMHPs, the observed behavior in 2DHPs points to a fundamentally different mechanism of light-induced halide redistribution, which is driven by site-selective occupation and local bonding energetics unique to the layered architecture. Notably, in all three mixed halide 2D perovskites with different halide ratios, we consistently observe a markedly higher PLQY after photoexcitation than in the initial phases. A possible explanation for the strong PLQY enhancement observed in the photoexcited phases is an efficient exciton-funneling process: photogenerated excitons in unswitched, wider-bandgap regions efficiently transfer into switched, narrower-bandgap regions via preferred hole funneling, where they recombine radiatively (Figure S23). We note that for this new excitonic mixed halide layered material system, future work combining both theoretical modeling and experimental characterization will be needed to elucidate the photoisomerization mechanism. In addition, the photoinduced switched phase exhibits a 3-fold increase in average exciton lifetime relative to the original phase (Figure S24). The concurrent enhancements in PLQY and exciton lifetime strongly indicate that halide isomerization within the PbX_6_^4–^ octahedra can substantially influence their optoelectronic properties.

To understand the onsets of the ion-switched states, we analyzed the full width at half-maximum (fwhm) of the stabilized PL spectra at various excitation intensities (Figures S20-S22). All three MHP compositions show broad fwhm at low excitation intensities suggesting that the mixed halide structures preserve stronger exciton-lattice interactions and less lattice ordering at room temperature (RT).^24^ This broadening of the PL spectra for the MHP compositions is consistent with broadband THz time-domain spectroscopy results (Figure S25),^24^ which reveal prominent signatures at ∼2 THz of phonon modes involving twisting and stretching of the Pb-halide octahedra.^25,26^ We observe a decay and broadening of these THz phonon signatures for the mixed halide species, which could be due to the intrinsically less crystalline and more disordered structures, with a random coexistence of Br- and I-related phonons. Note that such Pb-halide octahedron phonon modes can be involved in exciton–phonon interactions, promoting polaron formation and charge localization, especially under photoexcitation.^27^ A consistent narrowing in the PL fwhm is observed with increasing excitation intensity. This trend is observed across all three MHP compositions (Figure S26) and is absent in BA_2_PbI_4_ (Figure S27). Such PL peak narrowing with increasing excitation intensities at room temperature correlates with the transition from self-trapped excitons to free excitons, which dominate the exciton recombination by a narrow and sharp emission band at temperatures below 100 K (Figure S28).

Finally, we seek insights into the electronic band structure evolution induced by halide switching for different halide ratios. DFT calculations reveal a consistent reduction in the bandgap upon full halide reordering across all three mixed halide compositions. For Br_3_I, even the switching of a single I^–^ from the T-site to B-site can induce a significant bandgap narrowing of ∼0.4 eV, which highlights the sensitivity of optoelectronic properties to halide site occupancy. Bandgap reductions for Br_2_I_2_ and BrI_3_ were found to be ∼0.5 and ∼0.38 eV, respectively. Details of electronic band structures for Br_3_I and BrI_3_ before and after ion switching are shown in Figures S29–S30. These theoretical trends are corroborated by experimental optical bandgap values inferred from spatially resolved absorption maps (Figures S31–S32), which show consistent reductions of 0.07 eV (Br_3_I), 0.10 eV (Br_2_I_2_), and 0.10 eV (BrI_3_). A comparison of bandgap changes from DFT simulation and optical measurement is presented in Figure S33. The smaller reduction in optical bandgaps in comparison with DFT results is attributed to the partial nature of photoisomerization in the experiments, while DFT calculations assume fully switched configurations. These results support the idea that effective photoisomerization in 2DHPs requires the presence of chemically distinct halides occupying different crystallographic sites.

## Discussion

Photoisomerization offers a powerful, additive-free approach for light-driven reversible modulation of material properties with high spatial and temporal resolution, making it a compelling mechanism for applications in optical switching, data storage, and emerging quantum or neuromorphic technologies.^28,29^ In this work, we demonstrate that photoisomerization can be intrinsically activated in 2D mixed halide perovskites (2DMHPs), without relying on external dopants or composite systems. Our investigation of single-crystalline BA_2_PbBr_*x*_I_4–*x*_ (*x* = 1–3) 2DMHPs reveals that unlike the photoinduced halide segregation commonly observed in 3D perovskites,^14,15^ the 2D framework supports a fundamentally different light-responsive mechanism. Specifically, we uncover a site-selective halide switching process, in which Br^–^ and I^–^ ions exchange positions between distinct crystallographic sites—T-site and B-site—within individual PbX_6_^4–^ octahedra while avoiding long-range ion migration or phase separation.

By combining hyperspectral imaging, in situ X-ray diffraction, and density functional theory, we demonstrate that this photoisomerization induces a reversible lattice reconfiguration and substantial bandgap modulation, which offers structurally encoded, reversible control over their optoelectronic properties. We further show that the efficiency and threshold of this transformation are highly dependent on halide composition, and iodide-rich crystals exhibit significantly lower photoactivation thresholds. This composition-sensitive behavior highlights the role of the local halide environment and lattice polarizability in facilitating efficient light-induced switching. Altogether, these findings establish the halide site occupancy as a powerful structural lever for tuning the optoelectronic response of 2D perovskites under light.

Beyond elucidating the intrinsic photophysics of 2DMHPs, this work introduces a design principle for achieving reversible, homogeneous, and composition-tunable photoresponses in layered perovskites. The ability to control ion positions with light without compromising crystalline order opens promising directions for reconfigurable photonic architectures, solid-state optical memory, and quantum optoelectronic devices.

## Methods

### Materials

Dimethylformamide (DMF; 99.8%), gamma-butyrolactone (GBL; 99%), lead bromide (PbBr2; 99.999%), and lead iodide (PbI_2_; 99.999%) were purchased from Sigma-Aldrich. *n*-Butylammonium bromide (BABr; 99%) and *n*-butylammonium iodide (BAI; 99%) were purchased from Greatcell Solar Materials. All chemicals were used without further purification.

### Fabrication of 2DHP Single Crystals

All 2D perovskite single crystals were grown using a modified space-confining method.^18^ Briefly, 2D perovskite precursor solutions were prepared by dissolving stoichiometric quantities of the perovskite precursor powder in corresponding solvents (pure iodide-based perovskites were dissolved in GBL; pure bromide and all the mixed halide perovskites were dissolved in DMF) to obtain solutions with a concentration of 0.8 mol/L. After full dissolution, a drop of the solutions (20 μL) was drop-casted onto a precleaned glass substrate or silicon substrate. Then, a precleaned glass cover slide was quickly placed on top of the solution. The solution immediately spread between the two substrates. The substrate sets were then placed on a hot plate at room temperature, with the temperature of the hot plate gradually increasing to 80 °C at a rate of 1 °C/min. The temperature was maintained at 80 °C for 3 h and then gradually cooled to room temperature at a rate of 10 °C/h. After cooling, the substrate sets were stored in a dry air box (20 °C) for 3–5 days. Submillimeter-sized single crystals with thicknesses around 600 nm for glass substrates and 100–300 nm for silicon substrates were then isolated for characterization.

### Single-Crystal X-ray Diffraction Analysis

Single-crystal diffraction studies were carried out on the MX2 beamline of the Australian Synchrotron with monochromatic radiation of wavelength 0.71073 Å.^30^ The crystal was maintained at each given temperature in an open-flow nitrogen cryostream during data collection. The measurements of BA_2_PbBr_*x*_I_4–*x*_ were carried out at 100 K.

The measurement of BA_2_PbBr_2_I_2_ under illumination was performed at 100, 200, and 293 K to capture the halide swapping changes. The illumination was achieved by using a 405 nm LED light with an output intensity of around 50 mW/cm^2^. The light was focused onto the sample through a liquid light guide. The crystal was illuminated for 10 min at 100 K and then the data was collected. The crystal was then warmed to 200 K and illuminated for 20 min, and then data was collected. The crystal was illuminated for another 60 min and more data collected. An additional experiment was carried out in which a crystal was warmed to 293 K and data collected, then illuminated for two 20 min intervals, with data sets collected between. This gave data sets with 0, 20, and 40 min of illumination time. All data was collected with an Eiger 16 M detector, and the raw data was processed with the XDS software package.^31^ Initial solutions were obtained with SHELXT,^32^ and data was refined with SHELXL^33^ using the Olex2 interface.^34^ For each of the mixed halide systems, the halides were modeled as disordered, such that the overall occupancy was restrained to the expected Br:I ratio (1:3, 2:2, 3:1) but were modeled as distributed over the terminal and bridging positions.

### Hyperspectral Photoluminescence and Absorptance Microscopy

Spectrally resolved maps were acquired using an IMA hyperspectral microscope (Photon Etc.). For photoluminescence maps, 405 nm wide-field continuous-wave laser excitation was used. The detection wavelength was spectrally scanned using a volume Bragg grating with monochromatic images acquired successively at ∼2 nm spectral resolution. At each detection wavelength, the system performed automatic refocusing of the sample stage. The volume Bragg grating dispersed the light onto a 1392 × 1040 pixel silicon CCD detector with thermoelectric cooling. For absorptance maps, we first measure reflectance and transmittance maps individually before processing to obtain the absorptance.

### PL Spectra and Time-Resolved Confocal Photoluminescence Microscopy

We acquired PL spectra before and after light soaking or relaxation, as well as time-resolved photoluminescence lifetimes, using a PicoQuant MicroTime 200 time-resolved confocal photoluminescence microscopy setup. We used a 404 nm pulsed laser excitation (LDH-D-C-405, PicoQuant, pulse width ∼100 ps) at a repetition rate of 20 or 40 MHz, with measured laser power at the sample of 119 or 281 nW, respectively. We used a 20× air objective (Olympus Plan N, NA 0.4) for creating a focused excitation beam on the sample. The emission signal was separated from the excitation light by using a dichroic mirror (zt405rdc, Chroma), with an additional 425 nm long-pass filter and a 75 μm pinhole placed in the collection path. The PL signal was either focused onto a PMA Hybrid-42 detector (Picoquant) for single-photon counting (with a time resolution of 25 ps), or the spectra were acquired using an Andor Kymera 193i spectrograph.

### High-Resolution XRD Measurement

HRXRD was conducted on an Empyrean (Panalytical) 2 Bragg–Brentano geometry X-ray diffractometer with a Cu Kα1 source. A step size of 0.004° and an acquisition time of 0.5 s were employed. For in situ light soaking, a 385 nm LED light (Thorlabs M385LP1) with *f* = 20 mm collimating lens was driven under a current of 0.9 A and placed 4 cm on top of the sample.

### Widefield Fluorescence Microscopy

Widefield microscopic measurements were performed on an inverted Nikon microscope (Eclipse Ti2) coupled to a Nikon D610 for imaging measurements. A high-intensity mercury lamp coupled with a 405 nm band-pass filter was used as the excitation light source. The excitation light was delivered to the input of the objective with a single-mode optical fiber. Emission spectra were recorded using an Andor Kymera 328i spectrometer coupled to the output of a Nikon Ti2 microscope.

### Atomic Force Microscopy

AFM measurements were conducted using an Asylum Research MFP-3D Atomic Force Microscope. All measurements were performed in the standard tapping mode with AC160TS-R3 cantilevers from Oxford Instruments.

### Cathodoluminescence Spectroscopy

CL measurements were performed using a FEI Nova NanoSEM 450 FEGSEM and a Delmic SPARC Cathodoluminescence System with a Gatan C1003 liquid nitrogen cooling stage. An electron beam with a 2 kV high voltage, 40 μm objective aperture, and spot sizes ranging from 3 to 6.5 (resulting in a probe current of 49 pA to 3 nA) was used to gain sufficient CL intensity. To minimize electron beam damage, the sample was first navigated to the target position at low magnification. The region of interest was then approached without direct electron imaging, and the electron beam was “turned off” using the SEM beam blank function. To further reduce the electron dose, the electron beam was defocused to a disk of ∼10 μm in diameter. CL spectra were acquired in spot mode and continuously monitored over time to observe any changes. The electron beam was “unblanked” after the CL acquisition began, allowing for analysis of the evolution of CL spectra from the very beginning of electron exposure. For CL acquisition at cryo temperatures, the sample grown on a Si substrate was mounted on the cold stage by using a cryocompatible silver paste. The stage was first cooled to 93 K and held for 30 min to stabilize before CL acquisition. Then, the temperature was raised in steps to monitor the CL response versus the temperature. For each step, the temperature was held for 15 min after the temperature change and before CL acquisition.

### First-Principles Density Functional Theory (DFT) Calculations

We performed the DFT calculations using the projector augmented wave (PAW) method as implemented in the Vienna Ab initio Simulation Package (VASP).^35^ The PAW pseudopotentials were used to treat the effective interactions between the valence and frozen-core electrons. We used the generalized gradient approximation (GGA) within Perdew–Burke–Ernzerhof (PBE) scheme for the exchange-correlation term.^36^ We selected 12 × 12 × 1 gamma-centered k-points and 400 eV cutoff energy for structural optimization and electronic structure calculations. In these calculations, we set stringent convergence criteria, i.e., 10^–8^ eV for energy convergence and 0.01 eV/Å for force convergence. As the PBE functional cannot describe self-interactions correctly, we also calculated the electronic structures using the single-shot GW method.^37,38^ We used 2 × 2 × 1 gamma-centered k-points and 315 eV cutoff energy in these calculations. We postprocessed some of the VASP data using VASPKIT^39^ and AMSET^40^ and visualized the charge density and crystal structures by VESTA. The activation energy was calculated from the height of the energy profile using the nudged elastic band (NEB) method^41^ in VASP (with 4 × 4 × 1 k-points and force convergence of 0.06 eV/Å). The pre- and postprocessing were performed using the VTST tool.^42^

For the calculation of the minimum energy path (MEP), the total force acting on each image along the path is decomposed into two components: the spring force projected along the local tangent direction (*F*_*i*_^S^) and the true force derived from the energy gradient, projected perpendicular to the path (−∇*E*(*R_i_*)|_⊥_). Mathematically, the total force on each structure is decomposed as:

$$F_{i}=F_{i}^{S}{|}_{||}-\nabla E\left(R_{i}\right){|}_{⊥}$$

where the spring force along the path is given by:

FiS|||=k(|Ri+1−Ri|−|Ri−Ri−1)|)×τ^i

and the perpendicular component of the energy gradient is defined as:

∇E(Ri)|⊥=∇E(Ri)−∇E(Ri)×τ^i

Here, τ^i represents the normalized local tangent in the *i*-th phase. The value of the spring constant *k* is −5 eV/Ang^2^. The energy of each optimized structure along this path was evaluated by using first-principles electronic structure calculations.

### THz Time-Domain Spectroscopy

Measurements were performed at room temperature in a low-humidity (<1%) nitrogen gas environment. We measured the complex transmission T̃(ω) of a THz waveform through the 2DHP’s of interest (spin-coated on a quartz substrate) as a function of frequency ω/2π:

$$\mathrm{T̃}\left(\omega \right)=\frac{{Ẽ}_{\mathrm{sample}}\left(\omega \right)}{{Ẽ}_{\mathrm{ref}}\left(\omega \right)}$$

Here, ${Ẽ}_{\mathrm{sample}}\left(\omega \right)$ is the Fourier transform of the time-dependent THz waveform electric field *E*_sample_(*t*) transmitted through the 2DHP, ${Ẽ}_{\mathrm{ref}}\left(\omega \right)$ is the Fourier transform of the reference time-dependent THz waveform electric field *E*_ref_(*t*) transmitted through the bare quartz substrate (i.e., without the 2DHP). We can then calculate the real part of the THz conductivity, σ_real_(ω), of the 2DHP as a function of frequency ω/2π via:

$$\sigma_{\mathrm{real}}\left(\omega \right)=-\frac{\varepsilon_{0}c}{d}\left(n_{\mathrm{sub}}\left(\omega \right)+1\right)\left(\left|\mathrm{T̃}\left(\omega \right)\right|-1\right)$$

where ε_0_ is the vacuum permittivity, *d* is the sample thickness (measured via profilometry for each individual thin film), *c* is the speed of light in vacuum, and *n*_sub_ is the real part of the quartz substrate refractive index (retrieved via a separate measurement). We measured T̃(ω) at three different locations on each sample and reported the average σ_real_(ω), with error bars corresponding to the standard deviation. We generated the probing THz waveforms via optical rectification of laser pulses (central wavelength ∼850 nm; duration ∼30 fs; pulse energy 4.5 μJ) focused (off-axis parabolic mirror with 8 in. focal length) onto a 200 μm thick gallium phosphide (GaP) crystal. These femtosecond laser pulses were produced by a hybrid optical parametric amplifier (Light Conversion Orpheus-F) seeded with a Yb:KGW laser (Light Conversion Carbide, central wavelength ∼1030 nm, pulse duration ∼250 fs, pulse energy ∼125 μJ). The generated THz waveform electric field was measured in the time-domain via electro-optic sampling using a 200 μm thick GaP crystal, with THz peak electric fields of ∼900 V/cm (enabling high signal-to-noise ratio measurements of T̃(ω) ) and broad bandwidth THz waveform Fourier components from ∼0.5 to ∼7.5 THz. The focused THz waveform spot size at the sample position was ∼1 mm in diameter.

## Supplementary Material



## Data Availability

Data Supporting this paper are available at the University of Cambridge Apollo repository with the link as doi.org/10.17863/CAM.124673.

## Notes

SCXRD data, surface morphology and height profile, SEM EDX, TEM EDX, halide distribution, Monte Carlo simulations, steady-state CL and PL spectra comparison, hyperspectral PL imaging, in situ optical microscopic images, Halide switching and recovery measurement, PL spectra, TRPL measurement, THz conductivity, PL FWHM changes, calculated electronic band structures, spatial absorptance measurement, and comparison of DFT-calculated electronic and optical bandgap changes (PDF)
