# Supplementary material for: Light-Driven Intraoctahedral Halide Isomerization in Two-Dimensional Mixed Halide Perovskites
Source: J Am Chem Soc. 2026 Jan 13;148(3):3051–62. doi: 10.1021/jacs.5c15542 (PMC12856884; doi:10.1021/jacs.5c15542)
Supplement: Supplementary file 1 [file ja5c15542_si_001.pdf]

Supplementary materials for

Light-driven intra-octahedral halide isomerization in two-dimensional mixed halide perovskites

Wenxin Mao*[1, 2], Enamul Haque[3], Stephanie A. Bird[4], Milos Dubajic[1], Yang Lu[1], Xian Wei Chua[1], Zhou Xu[5], Xinjuan Li[6], Mitko Oldfield[6], Jialu Li[7], Wenqi Yan[2], Christopher R. Hall[7], Qingdong Lin[2], Jie Zhao[2], Anthony S. R. Chesman[8], Gary Beane[6], Agustin Schiffrin[6], Caterina Ducati[6], Nikhil Medhekar[3], Samuel D. Stranks*[1] and Udo Bach*[2].

Affiliation:

1 Department of Chemical Engineering and Biotechnology, University of Cambridge, Philippa Fawcett Drive, Cambridge, CB3 0AS, UK

2 ARC Centre of Excellence in Exciton Science, Department of Chemical and Biological Engineering, Monash University, Clayton, Vic 3800, Australia

3 ARC Centre of Excellence in Future Low-Energy Electronics Technologies, Department of Materials and Science Engineering, Monash University, Clayton, Vic 3800, Australia

4 Australian Synchrotron, Clayton, Vic 3168, Australia

5 Monash Centre for Electron Microscopy, Monash University, Vic 3800, Australia

6 Department of Materials Science and Metallurgy, University of Cambridge, Cambridge, CB3 0FS UK

7 ARC Centre of Excellence in Future Low-Energy Electronics Technologies, School of Physics and Astronomy, Monash University, Clayton, Vic 3800, Australia

8 ARC Centre of Excellence in Exciton Science, The University of Melbourne, Vic 3010, Australia

9 CSIRO Manufacturing, Clayton, Vic 3168, Australia

*Correspondence authors. Email: wm362@cam.ac.uk; sds65@cam.ac.uk; udo.bach@monash.edu

Table S1. SCXRD data for $BA_2PbBr_2I_2$ measured at 100 K and 200 K with photoexcitation.

	$BA_2PbI_2Br_2$_100K_10minLED	$BA_2PbI_2Br_2$_200K_20minLED	$BA_2PbI_2Br_2$_200K_80minLED
Empirical formula	$C_8H_{24}Br_2I_2N_2Pb$	$C_8H_{24}Br_2I_2N_2Pb$	$C_8H_{24}Br_2I_2N_2Pb$
Formula weight	769.10	769.10	769.10
Temperature (K)	100(2)	200(2)	200(2)
Wavelength (Å)	0.71073	0.71073	0.71073
Crystal System	orthorhombic	orthorhombic	orthorhombic
Spacegroup	Pbca	Pbca	Pbca
a (Å)	8.3750(17)	8.4680(17)	8.4280(17)
b (Å)	8.2300(16)	8.3220(17)	8.2790(17)
c (Å)	27.677(6)	27.951(6)	27.869(6)
α (°)	90	90	90
β (°)	90	90	90
γ (°)	90	90	90
Volume ($Å^3$)	1907.7(7)	1969.7(7)	1944.6(7)
Z	4	4	4
Calculated density (g/cm^3)	2.678	2.654	2.627
Absorption coefficient (mm^{-1})	16.251	15.739	15.943
F(000)	1376.0	1376.0	1376.0
Crystal size (mm)	0.15 × 0.1 × 0.01	0.15 × 0.1 × 0.01	0.15 × 0.1 × 0.01
Theta range (º)	2.942 to 52.74	2.914 to 64.04	2.922 to 52.73
Reflections collected/unique	23494/1955	29365/3021	21310/1990
R_{int}	0.0886	0.1185	0.1406
Completeness to theta = 25 °	99.9	99.9	99.8
Data/restraints/parameters	1955/0/91	3021/0/90	1990/7/91
Final R indexes [I>=2σ (I)]	R_1 = 0.0423, wR_2 = 0.1112	R_1 = 0.0916, wR_2 = 0.3368	R_1 = 0.0908, wR_2 = 0.2777
Final R indexes [all data]	R_1 = 0.0454, wR_2 = 0.1142	R_1 = 0.1465, wR_2 = 0.3761	R_1 = 0.1205, wR_2 = 0.3111

Table S2. SCXRD data for $BA_2PbBr_2I_2$ measured at 293 K with and without photoexcitation.

Identification code	$BA_2PbBr_2I_2$_ 293K_0minLED	$BA_2PbBr_2I_2$_ 293K_20minLED	$BA_2PbBr_2I_2$_ 293K_40minLED
Empirical formula	$C_8H_{24}Br_2I_2N_2Pb$	$C_8H_{24}Br_2I_2N_2Pb$	$C_8H_{24}Br_2I_2N_2Pb$
Formula weight	769.10	769.10	769.10
Temperature/K	293(2)	293(2)	293(2)
Crystal system	orthorhombic	orthorhombic	orthorhombic
Space group	Pbca	Pbca	Pbca
a/Å	8.4190(17)	8.3960(17)	8.3740(17)
b/Å	8.2790(17)	8.2620(17)	8.2770(17)
c/Å	28.097(6)	28.027(6)	28.104(6)
α/°	90	90	90
β/°	90	90	90
γ/°	90	90	90
Volume/$Å^3$	1958.4(7)	1944.2(7)	1947.9(7)
Z	4	4	4
Calculated density (g/cm^3)	2.609	2.628	2.623
Absorption coefficient (mm^{-1})	15.831	15.946	15.916
F(000)	1376.0	1376.0	1376.0
Crystal size/mm^3	0.15 × 0.05 × 0.005	0.15 × 0.05 × 0.005	0.15 × 0.05 × 0.005
Theta range (°)	2.898 to 52.754	2.906 to 52.782	2.898 to 52.766
Reflections collected/unique	23916/2012	23805/1990	22762/2002
R_{int}	0.1093	0.1734	0.2670
Completeness to theta = 25 °	99.9	99.9	99.9
Data/restraints/parameters	2012/4/90	1990/3/66	2002/6/65
Final R indexes [I>=2σ (I)]	R_1 = 0.0772, wR_2 = 0.1923	R1 = 0.1111, wR2 = 0.2547	R_1 = 0.1160, wR_2 = 0.2770
Final R indexes [all data]	R_1 = 0.0838, wR_2 = 0.2006	R_1 = 0.1375, wR_2 = 0.2850	R_1 = 0.1834, wR_2 = 0.3337
GooF	1.090	1.000	0.948
Largest diff. peak/hole / e $Å^{-3}$	4.25/-2.52	7.16/-1.61	3.94/-1.03

Table S3. Occupancy summary for for $BA_2PbBr_2I_2$ measured at 293 K with and without photoexcitation.

Temperature	Illumination	I-Terminal ratio	Br-Terminal ratio	I-Bridging ratio	Br-Bridging ratio
100 K	0 min	0.681(11)	0.319(11)	0.319(11)	0.681(11)
293 K	0 min	0.60(2)	0.40(2)	0.40(2)	0.60(2)
293 K	20 min	0.57(3)	0.43(3)	0.43(3)	0.57(3)
293 K	40 min	0.36(3)	0.64(3)	0.64(3)	0.36(3)

Table S4. Single crystal X-ray diffraction (SCXRD) data for $BA_2PbBr_xI_{4-x}$ (x=0, 1, 2, 3, 4).

	BA_2PbBr_4	BA_2PbBr_3I	$BA_2PbI_2Br_2$	BA_2PbI_3Br	BA_2PbI_4
Empirical formula	$C_8H_{24}Br_4N_2Pb$	$C_8H_{24}Br_3IN_2Pb$	$C_8H_{24}Br_2I_2N_2Pb$	$C_8H_{24}BrI_3N_2Pb$	$C_8H_{24}I_4N_2Pb$
Formula weight	675.12	722.11	769.10	816.09	863.08
Temperature (K)	100(2)	100(2)	100(2)	100(2)	100(2)
Wavelength (Å)	0.71073	0.71073	0.71073	0.71073	0.71073
Crystal System	orthorhombic	orthorhombic	orthorhombic	orthorhombic	orthorhombic
Spacegroup	Pbca	Pbca	Pbca	Pbca	Pbca
a (Å)	8.2430(16)	8.2660(17)	8.3970(17)	8.4880(17)	8.4340(17)
b (Å)	8.1610(16)	8.1550(16)	8.2500(16)	8.3600(17)	8.9880(18)
c (Å)	27.560(6)	27.599(6)	27.784(6)	27.715(6)	26.275(5)
α (°)	90	90	90	90	90
β (°)	90	90	90	90	90
γ (°)	90	90	90	90	90
Volume ($Å^3$)	1854.0(6)	1860.4(6)	1924.7(7)	1966.6(7)	1991.8(7)
Z	4	4	4	4	4
Calculated density (g/cm^3)	2.419	2.578	2.654	2.756	2.878
Absorption coefficient (mm^{-1})	17.702	17.152	16.107	15.302	14.654
F(000)	1232.0	1304.0	1376	1448.0	1520.0
Crystal size (mm)	0.1 × 0.02 × 0.005	0.15 × 0.1 × 0.01	0.15 × 0.1 × 0.010	0.15 × 0.01 × 0.1	0.015 x 0.01 x 0.01
Theta range (°)	5.758 to 49.422	2.952 to 52.744	5.668 to 52.744	2.938 to 52.74	5.74 to 63.98
Reflections collected/unique	8471 / 1579	20828 / 1900	23674 / 1972	24251 / 2019	29503 / 2890
R_{int}	0.2771	0.1367	0.0887	0.0978	0.1033
Completeness to theta = 25 °	99.6%	99.9%	99.8%	99.9%	97.7%
Data/restraints/ parameters	1579/0/73	1900/37/85	1972/0/91	2019/26/78	2890/0/72

Final R indexes [I>=2σ (I)]	R_1 = 0.1183, wR_2 = 0.2777	R_1 = 0.0697, wR_2 = 0.2037	R_1 = 0.0380, wR_2 = 0.1042	R_1 = 0.0430, wR_2 = 0.1197	R_1 = 0.0554, wR_2 = 0.1654
Final R indexes [all data]	R_1 = 0.1278, wR_2 = 0.2894	R_1 = 0.0822, wR_2 = 0.2168	R_1 = 0.0404, wR_2 = 0.1095	R_1 = 0.0499, wR_2 = 0.1258	R_1 = 0.0598, wR_2 = 0.1746

Table S5. Occupancy summary for $BA_2PbBr_xI_{4-x}$ (x=0, 1, 2, 3, 4) measured at 100 K.

Structure	Compound	I-Terminal ratio	Br-Terminal ratio	I-Bridging ratio	Br-Bridging ratio
BA_2PbBr_3I	$C_8H_{24}Br_3IN_2Pb$	0.25	0.75	0.25	0.75
$BA_2PbI_2Br_2$	$C_8H_{24}Br_2I_2N_2Pb$	0.681(11)	0.319(11)	0.319(11)	0.681(11)
BA_2PbI_3Br	$C_8H_{24}BrI_3N_2Pb$	0.8	0.2	0.7	0.3

We note that in the Br_2I_2 structures we can refine freely and so get an error because they have a 1:1 halide ratio, but in the 1:3 and 3:1 structures it is not possible to do this in a way that guarantees that it will make chemical sense. Therefor the occupancy was refined freely and then fixed to occupancy that made chemical sense but was informed by the free refinement.

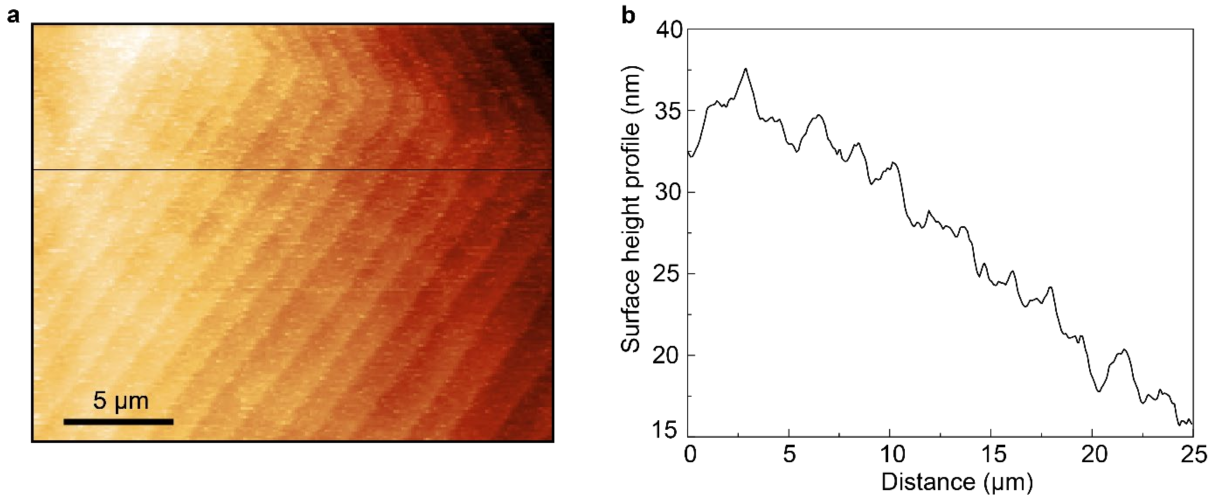

Figure S1. Surface morphology and height profile of a $BA_2PbBr_2I_2$ single crystal. (a) A representative AFM map of a $BA_2PbBr_2I_2$ surface. (b) Corresponding height profile along the black line in (a).

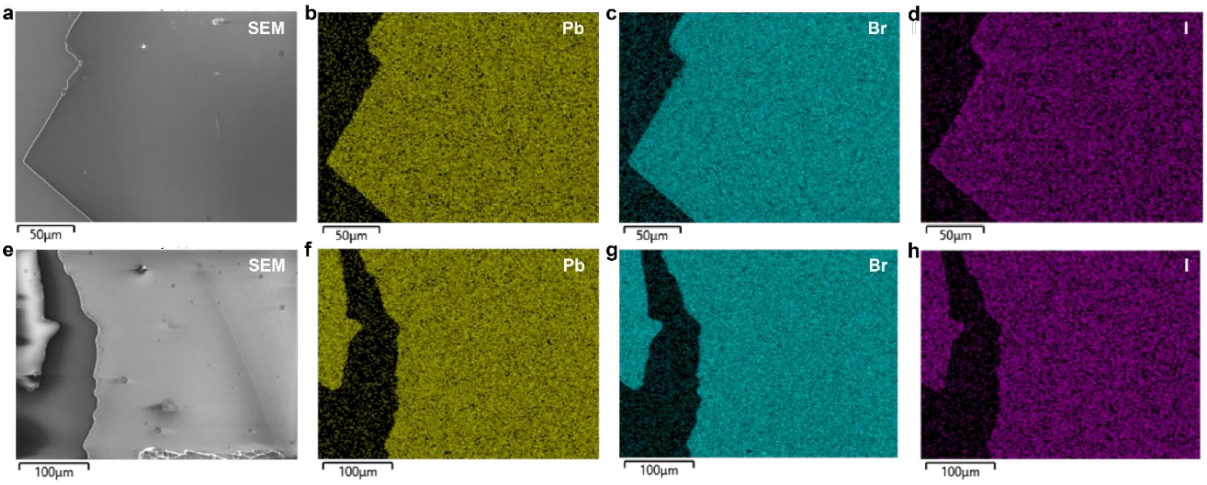

Figure S2. Large-area halide distribution analysis by SEM EDX mapping for two different crystals. (a, e) SEM images of two $BA_2PbBr_2I_2$ single crystals. (b-h) elemental maps of (b, f) Pb, (c, g) Br and (d, h) I.

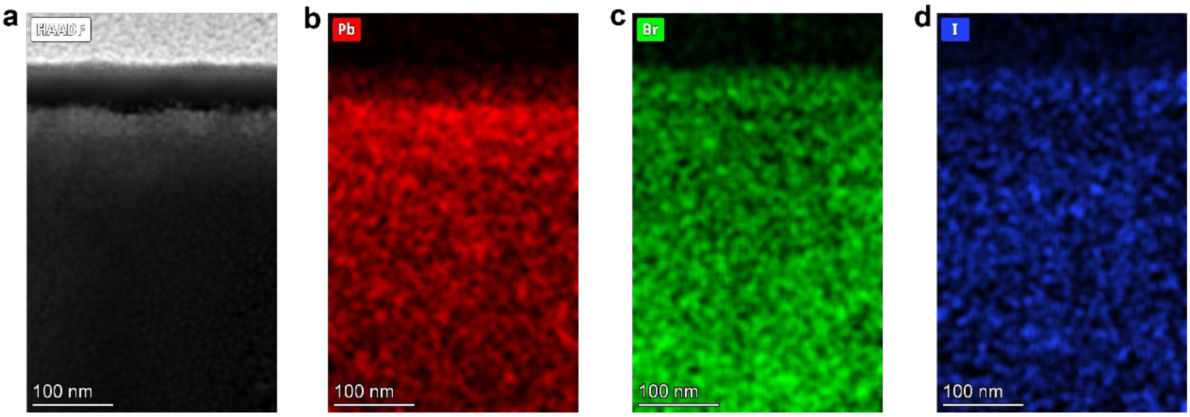

Figure S3. Vertical halide distribution analysis by TEM EDX mapping. (a) HAADF image of a cross sectional $BA_2PbBr_2I_2$ single crystal. (b-d) elemental maps of (b) Pb, (c) Br and (d) I. The cross-section sample was cut by FIB from a large single crystal.

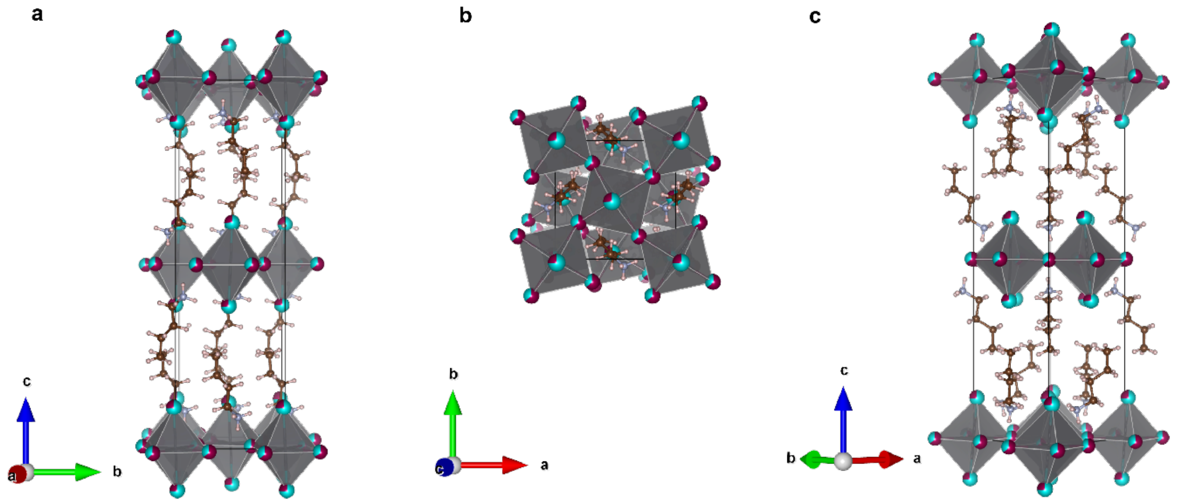

Figure S4. SCXRD result of $BA_2PbBr_2I_2$ measured at 100 K. (a-c) are unit cell plotted at different view directions. In this structure with equilibrium bromide and iodide ratio, 68% bromide occupies B-site and 68% iodide occupies T-site. Details of structural refinement are presented in Supplementary Table 1.

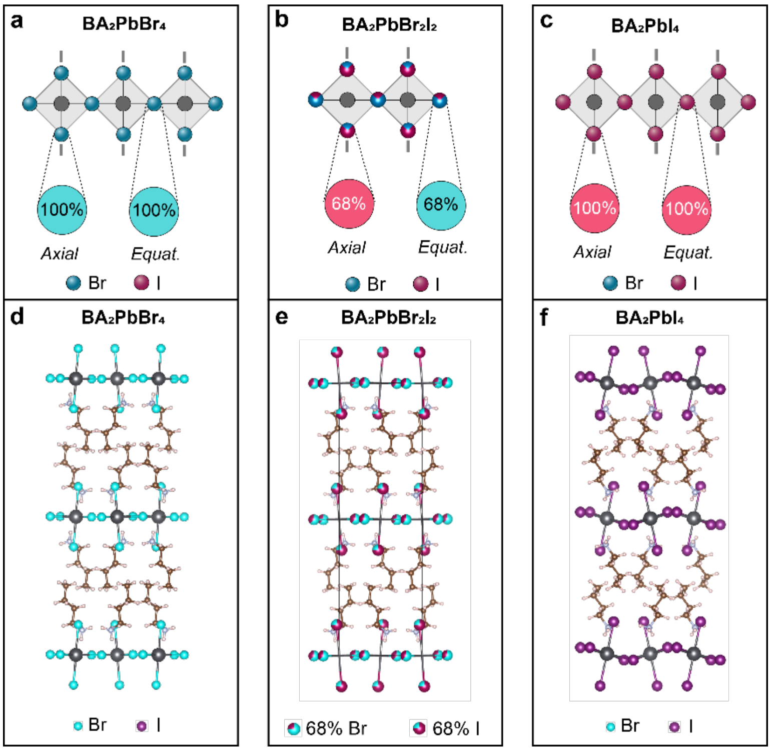

Figure S5. Halide distribution of BA_2PbBr_4, BA2PbBr₂I₂ and BA_2PbI_4 structures derived from SCXRD data. (a-c) Schematics showing the Br and I occupancy at B-site and T-site for different halide compositions. Corresponding crystal structures from SCXRD data measured at 100 K. The structures for BA_2PbBr_4 and BA_2PbI_4 structures are consistent with previous reports[1 2].

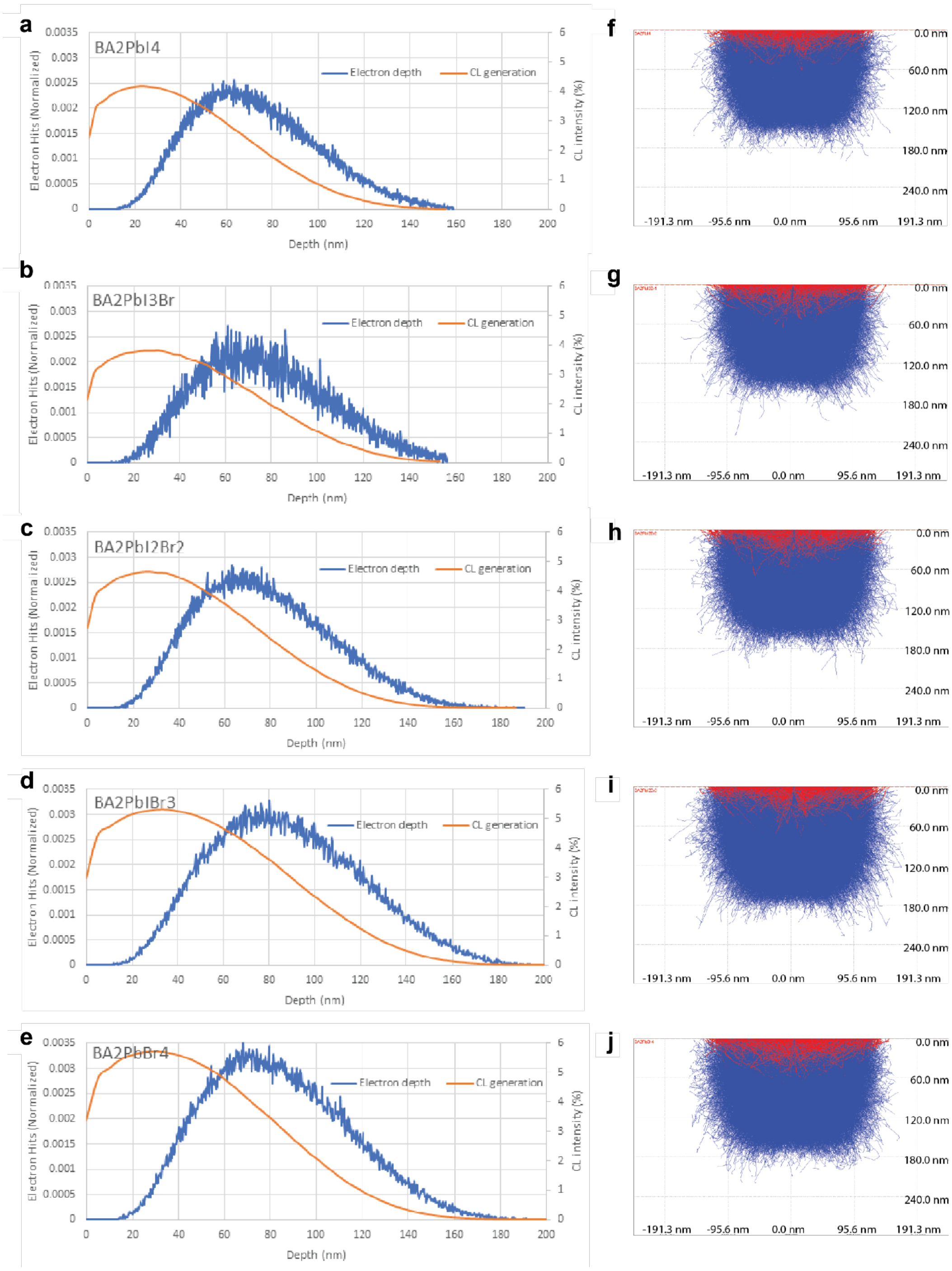

Figure S6. Monte Carlo simulations of electron penetration within $BA_2PbBr_xI_{4-x}$ single crystals with different halide compositions. (a-e) Simulated electron beam penetration depth profile (blue) and relative CL intensity generation as a function of depth (orange) with an indication where most CL emission is expected for each 2D perovskite composition. (f-j) Corresponding two-dimensional maps showing the spatial generation of CL signal within the materials. Note the average thickness of those crystals are 300 nm to 500 nm. Therefore, electron excitation in for CL measurements can represent a bulk excitation.

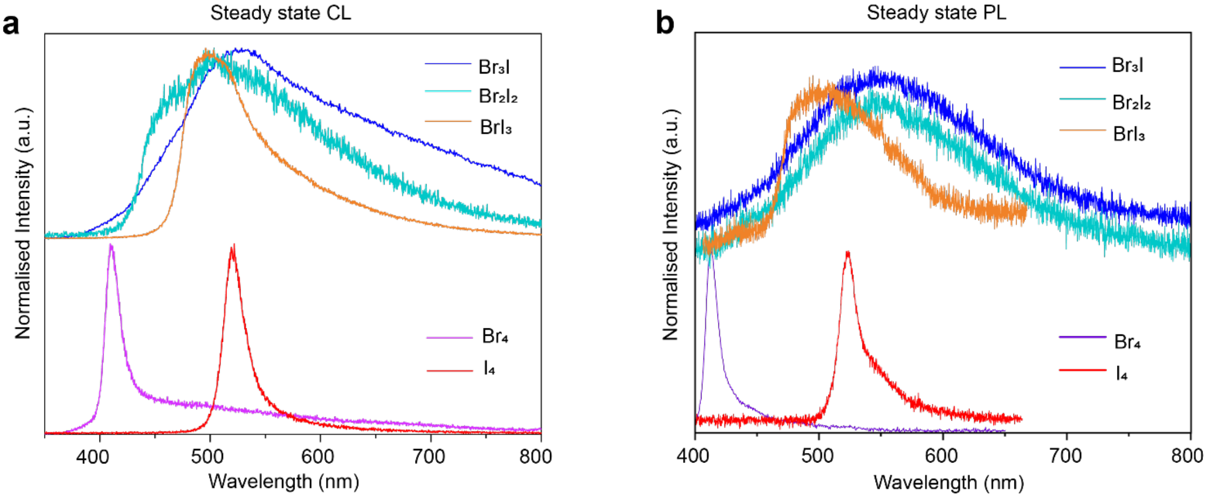

Figure S7. Steady state CL and PL spectra comparison for $BA_2PbBr_xI_{4-x}$. (a) Steady state CL emission spectra. (b) Steady state PL emission spectra. Both CL and PL results show similar spectrum features for all five halide compositions. In particular, single halide compositions (BA_2PbBr_4 and BA_2PbI_4) show sharp exciton emission while the mixed halide compositions have broad emission features in both CL and PL. Consistent exciton recombination behaviour in bulk excited CL and surface excited PL supports that the broad emission of the mixed halide 2D perovskite single crystals is an intrinsic feature rather than a surface trap states-related emission.

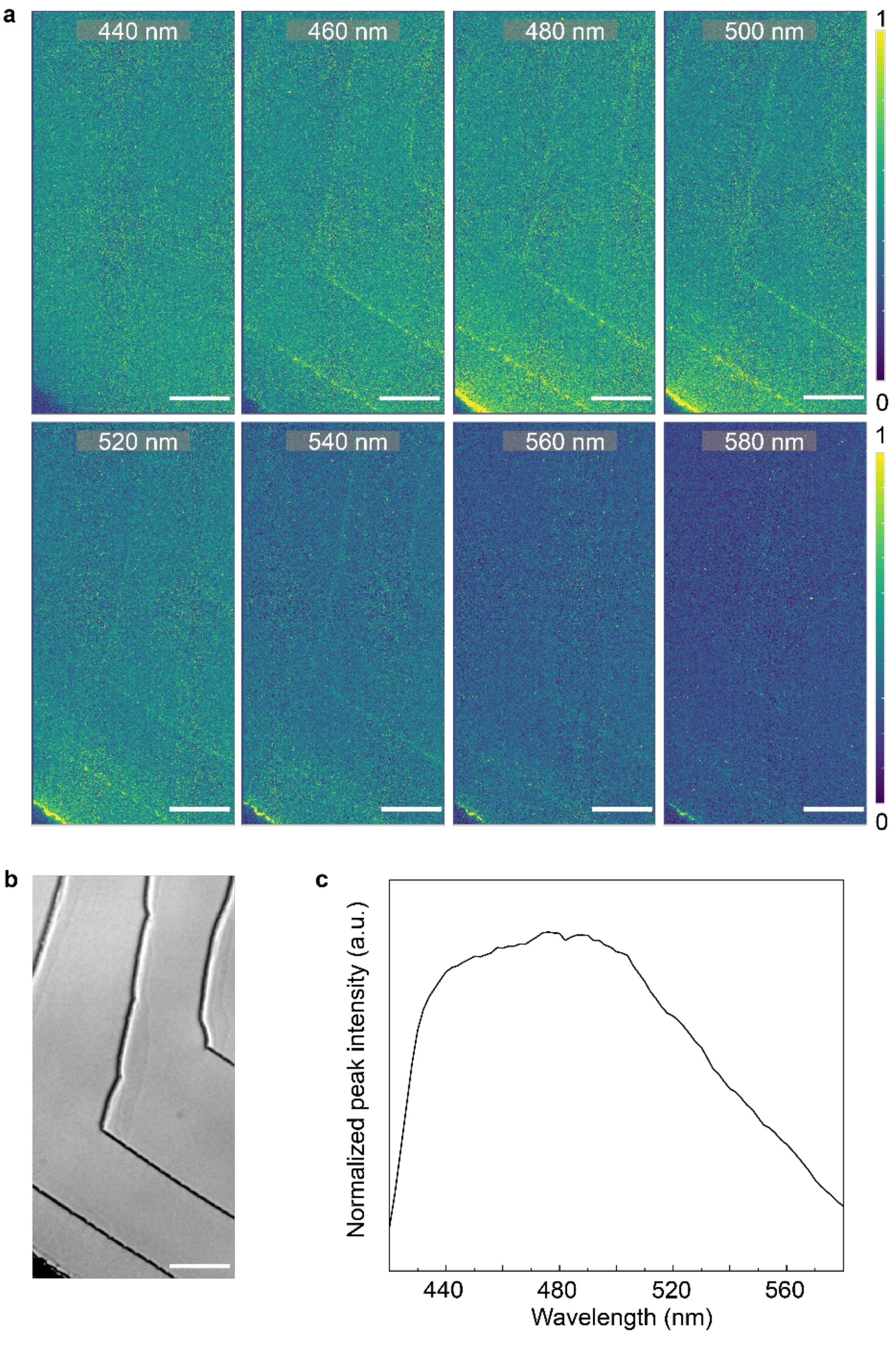

Figure S8. Hyperspectral PL imaging of a 2D $BA_2PbBr_2I_2$ single crystal before extended photoexcitation. (a) Hyperspectral PL maps of the thin $BA_2PbBr_2I_2$ single crystal at different central wavelengths. (b) Optical image of the thin $BA_2PbBr_2I_2$ crystal. The scalebars for all images are 20 μm. (c) An average PL spectrum of the above crystal.

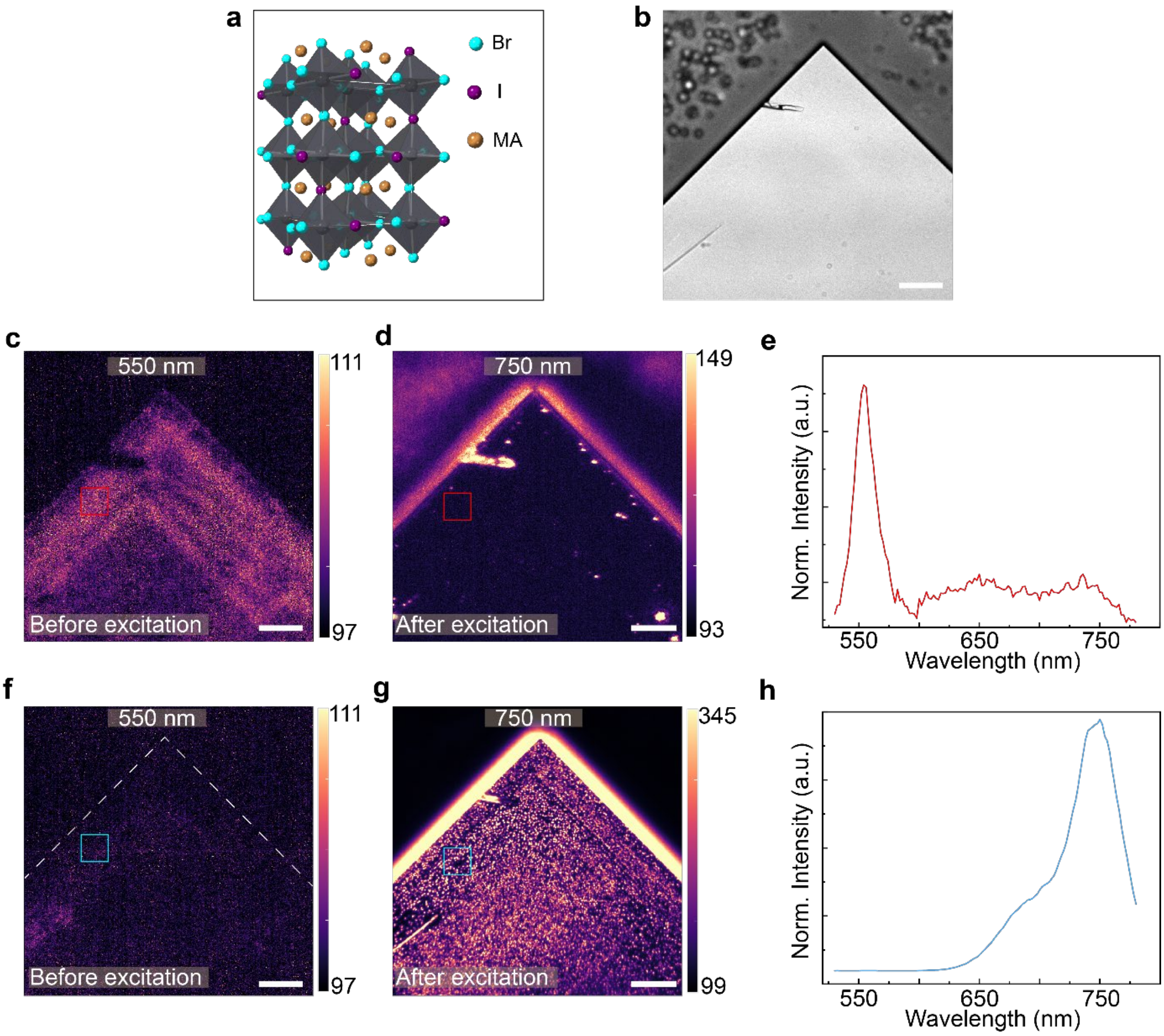

Figure S9. Hyperspectral PL imaging of a 3D $MAPbBr_xI_{3-x}$ single crystal. (a) Schematic illustration of the random halide distribution in a 3D mixed halide perovskite network. (b) Microscopic image of a $MAPbBr_xI_{3-x}$ single crystal platelet. The scalebar is 20 µm. (c-d) Hyperspectral PL maps before photoexcitation for the same crystal in (b), with central wavelengths of (c) at 550vnm for Br-rich phase and (d) at 750 nm for I-rich phase. (e) corresponding PL spectrum at a region of interest. (f-g) Hyperspectral PL maps after photoexcitation to a 405-nm CW laser with an intensity of ~80 mW/cm^2 for 5 min. The central wavelengths for (f) and (g) are the same as (c) and (d) for Br-rich and I-rich phases. (h) The PL spectra from the same ROI after photoexcitation. Before photoexcitation as shown in the hyperspectral PL image in (c), the Br-rich phase at 550 nm is homogeneous across the entire illuminated crystal while with no I-rich emission (d), demonstrating a uniform halide distribution before photoexcitation. The large emitting region presented in (d) is from a defect, which is presented in the optical image (b). In the PL spectra shown in (e), there are two weak emission peaks centred at 650 nm and 730 nm, which are intermediate halide segregation phases due to the ongoing halide segregation upon the continuous photoexcitation during the measurement, consistent with our previous phase segregation studies[3 4 5 6 7]. After 5 min light soaking, there is a significant decrease in Br-rich emission (f) while a much-enhanced emission from the I-rich phase. Notably, the I-rich phases are from individual segregated domains

spreading across the crystal. This is a key feature of photoinduced halide segregation in mixed halide single crystal studies.

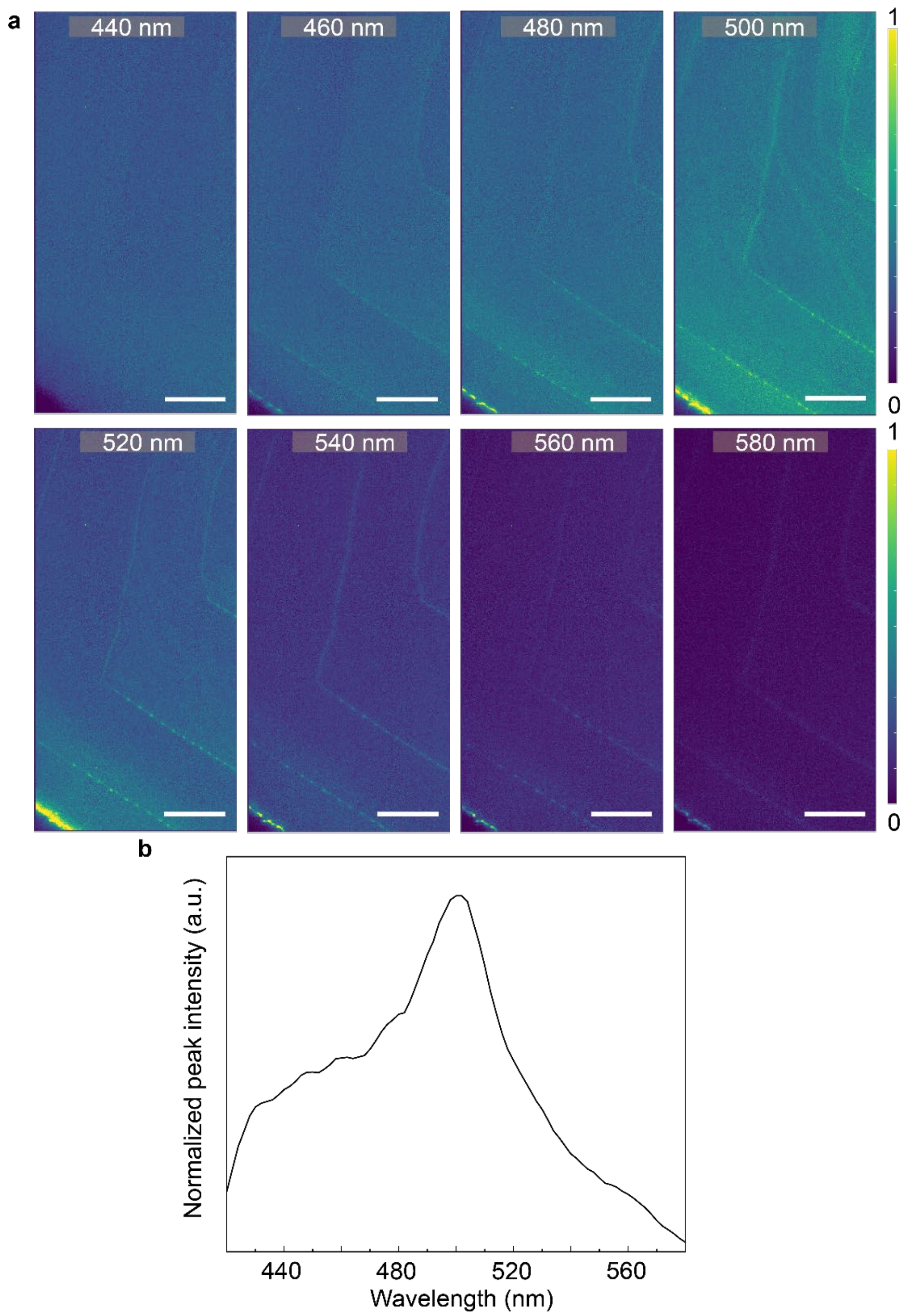

Figure S10. Hyperspectral PL imaging of a 2D $BA_2PbBr_2I_2$ single crystal after extended photoexcitation by a 405 nm CW laser with an excitation intensity of 80 mW/cm^2 for 5 min. (a) Hyperspectral PL maps of the thin $BA_2PbBr_2I_2$ single crystal at different central wavelengths. The scalebars for all images are 20 µm. (b) An average PL spectrum of the above crystal.

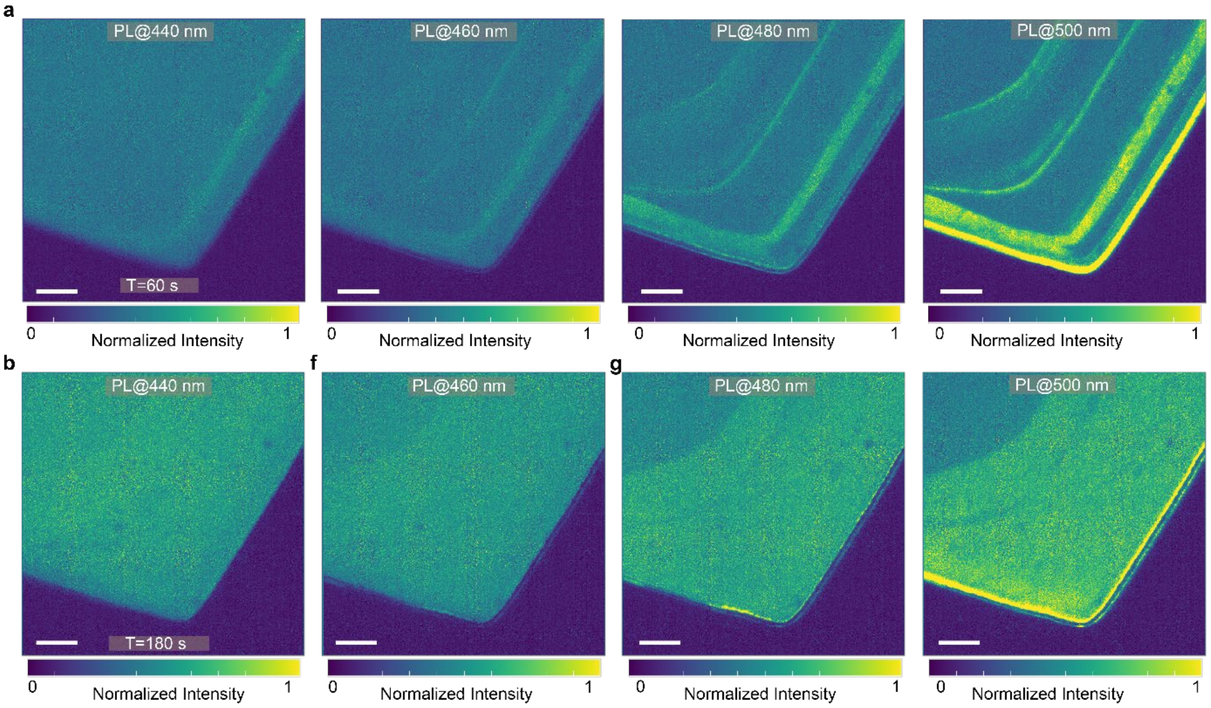

Figure S11. Hyperspectral PL imaging of a 2D $BA_2PbBr_2I_2$ single crystal after extended photoexcitation by a 405 nm CW laser with an excitation intensity of 80 mW/cm^2 for 60 s and 180 s. (a) Hyperspectral PL maps of the thin $BA_2PbBr_2I_2$ single crystal at different central wavelengths after extended photoexcitation for 60 s. (b) Hyperspectral PL maps of the thin $BA_2PbBr_2I_2$ single crystal at different central wavelengths after extended photoexcitation for 180 s. The scalebars for all images are 20 µm.

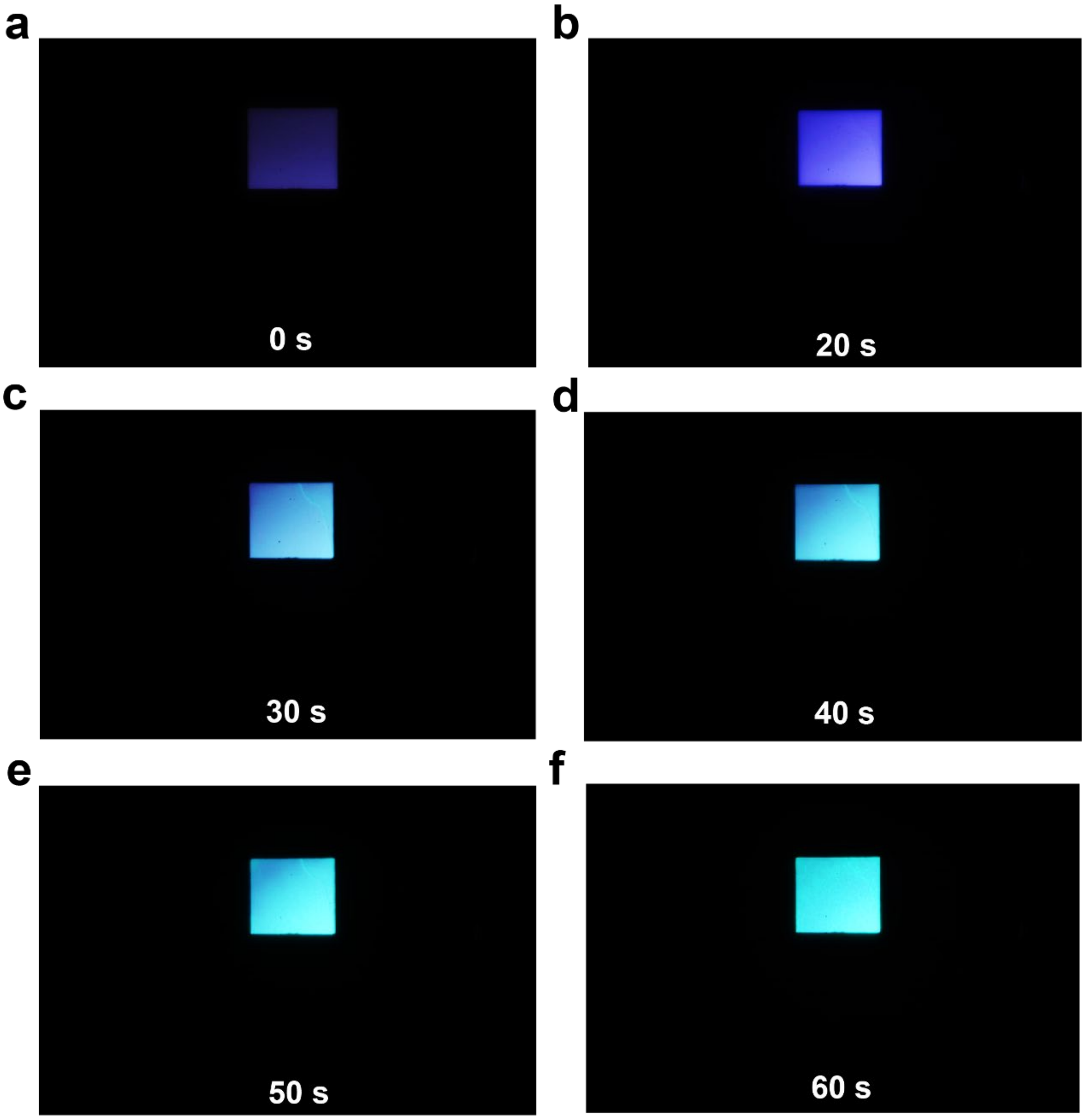

Figure S12. In situ photoluminescence microscopic images of $BA_2PbBr_2I_2$ under 405 nm CW light exposure. (a-f) Widefield photoluminescence microscopic images show PL change induced by halide isomerization. The central square area is the photo-excited area. The colour changes are real fluorescence change upon photoexcitation. No phase segregated emissions are observed.

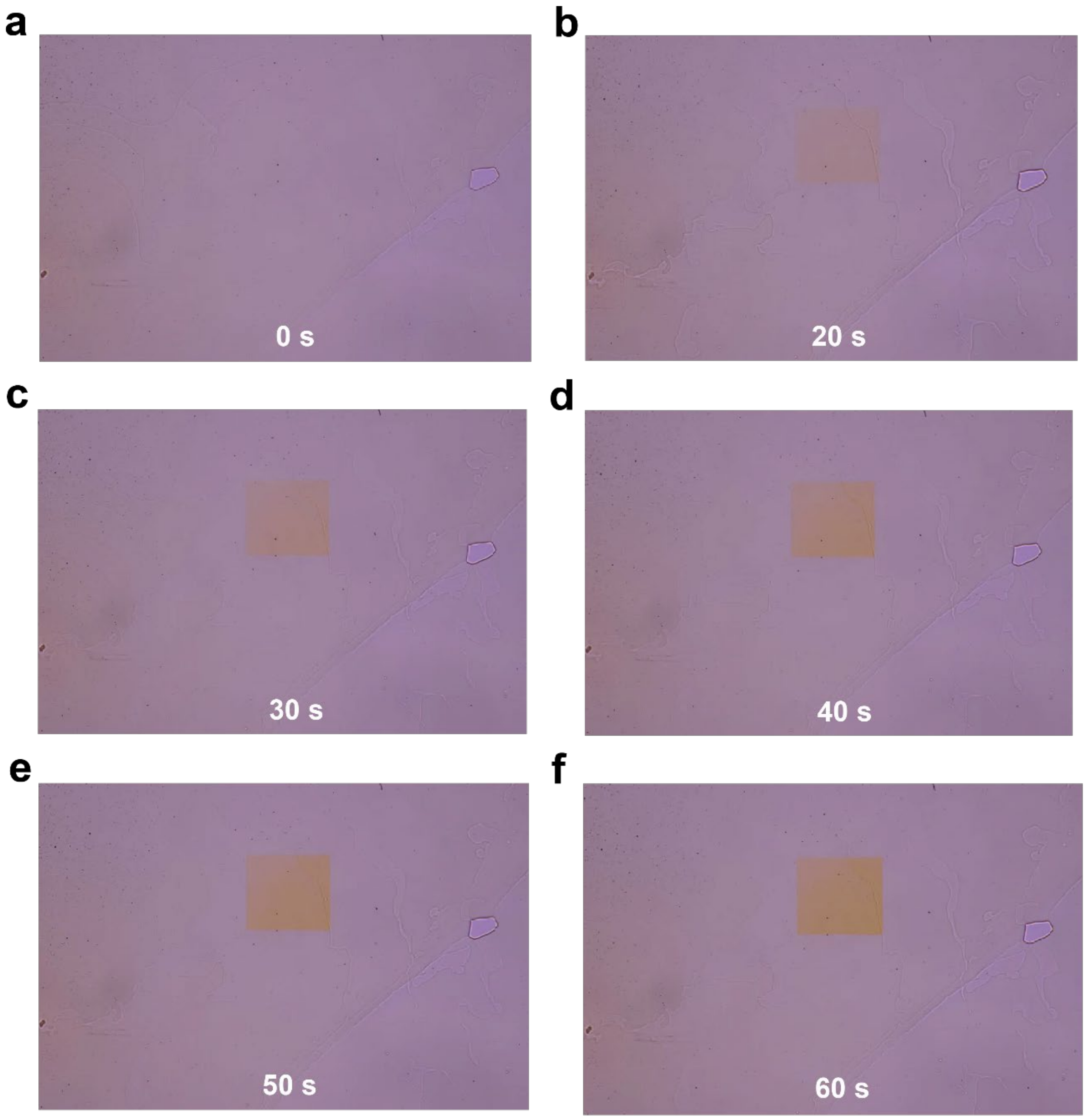

Figure S13. In situ optical microscopic images of $BA_2PbBr_2I_2$ under 405 nm CW light exposure. (a-f) Optical microscopy images show optical bandgap reduction induced by the halide switching. The central square area is the photo-excited area. A uniform distribution of the ion-switched phase covers the illuminated square region with no observation of segregated domains, consistent with previous PL imaging result.

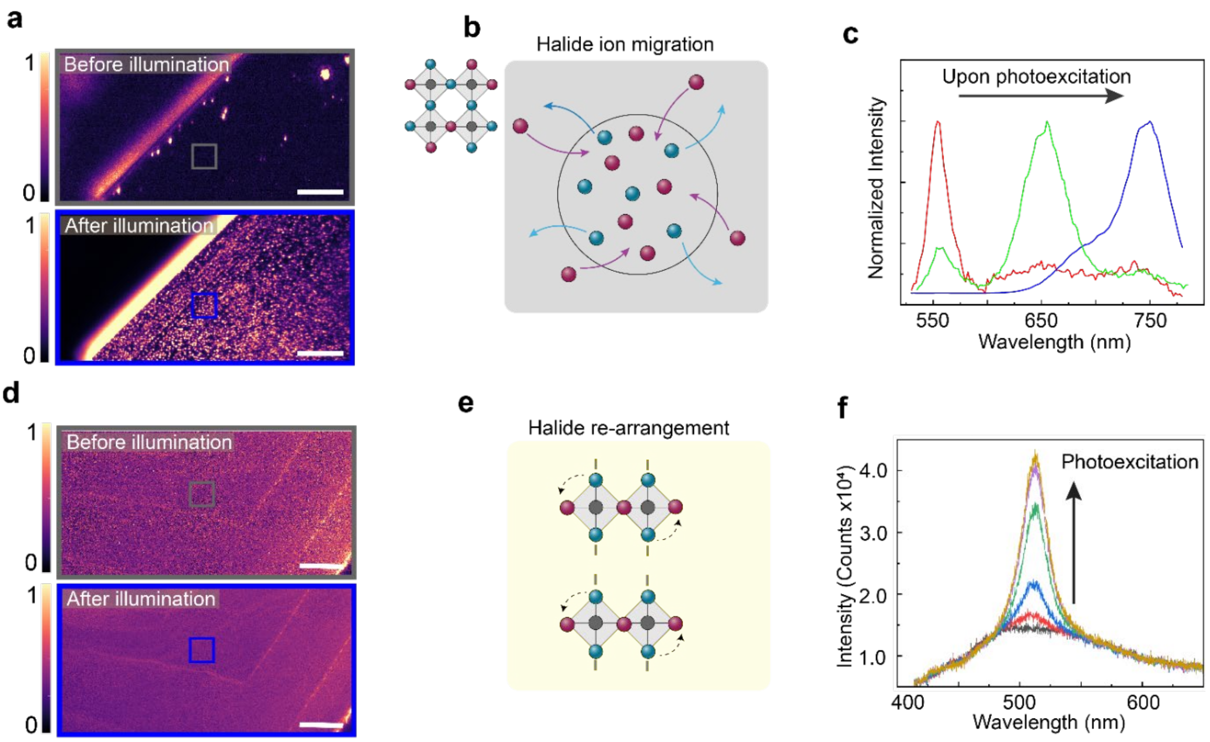

Figure S14. Mechanistic illustration for halide segregation for 3DMHP and halide switching for 2DMHP. (a) Hyperspectral PL maps of a thin $MAPbBr_xI_{3-x}$ single crystal. The top and bottom images represent before and after photoexcitation by a 405 nm CW laser with an excitation intensity of 80 mW/cm^2 for 5 min. The central wavelength of the PL maps is 750 nm, which is the iodide-rich phase wavelength window. (b) Schematic illustration of light induced halide segregation for 3D $MAPbBr_xI_{3-x}$. (c) PL spectra changes while under photoexcitation for a $MAPbBr_xI_{3-x}$. (d) Hyperspectral PL maps of a thin $BA_2PbBr_2I_2$ single crystal. The top and bottom images represent before and after photoexcitation by a 405 nm CW laser for 5 min. The central wavelength of the PL maps is 500 nm, which is the iodide-rich phase wavelength window. (e) Schematic illustration of photoisomerization for 2D $BA_2PbBr_2I_2$. (f) PL spectra changes while under photoexcitation for a 2D $BA_2PbBr_2I_2$.

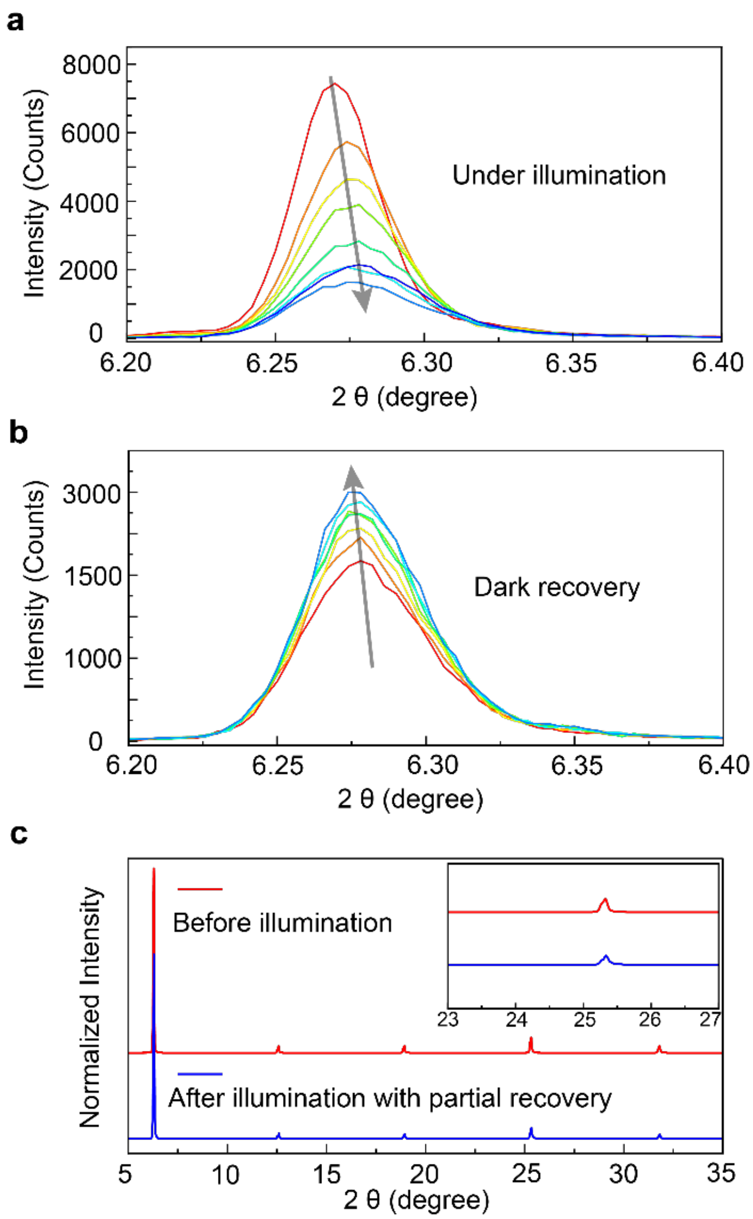

Figure S15. In situ crystal structure characterization of halide switching under photoexcitation and recover under the dark. Room temperature XRD of thin $BA_2PbBr_2I_2$ crystals grown on a glass substrate. (a) XRD diffraction pattern changes during photoexcitation. (b) XRD diffraction pattern changes under the dark. Excitation: 385-nm LED with intensity of 100 mW/cm^2. The crystals were illuminated for 60 s followed by a quick XRD scan at a small angle range to maintain a relatively high resolution in a short scan time (3 min). The total illumination time for 8 scans is 8 min. In the reversible measurement, the crystals were relaxed with LED off. A total of around 1-hour dark measurement with around 25 min for XRD scanning time included was applied in the reversible test. The major change in (a) includes a clear peak shifting to high 2θ and d reduction in peak intensity, demonstrating the progression of halide switching under photoexcitation. No phase segregation related peak splitting is observed. The major change in (b) includes a relaxation of both 2θ angle and diffraction intensity towards the original phase in the dark state. (c) Full XRD patterns of the above two highlighted XRD patterns shown in (a) and (b). The inset in (c) highlights the comparison of a diffraction peak at a higher angle.

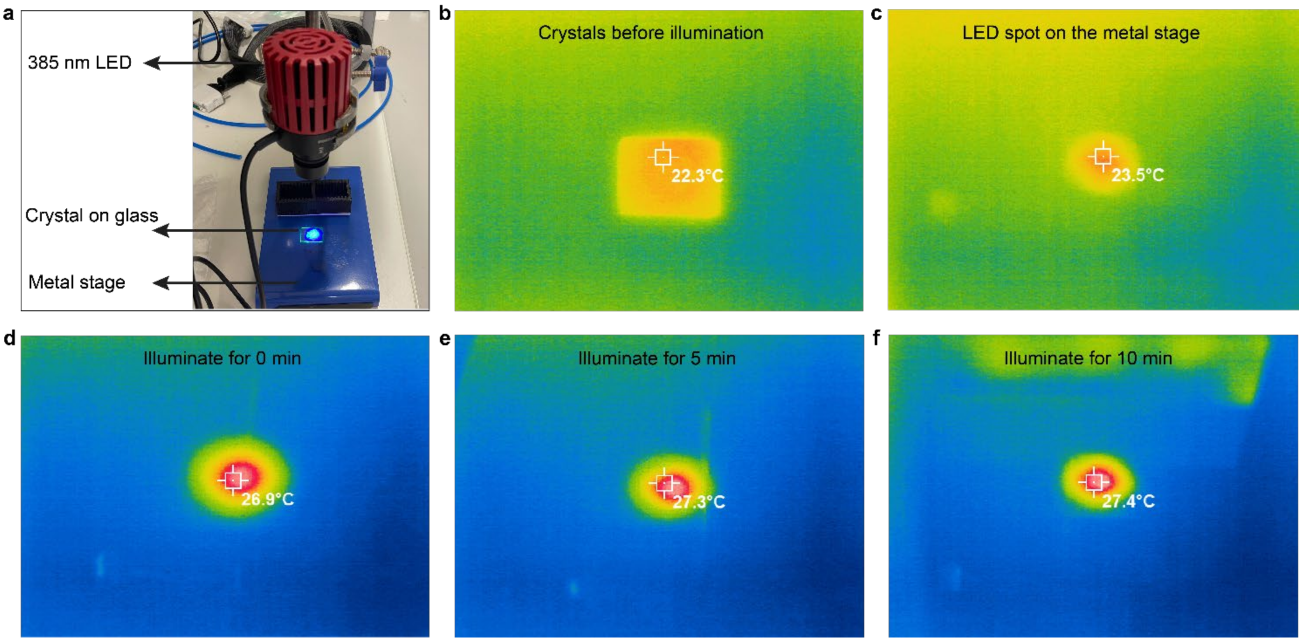

Figure S16. Light induced temperature change under photoexcitation. (a) Experimental setup: $BA_2PbBr_2I_2$ crystals were grown on a 0.2 mm thin cover slide and encapsulated by a 100 nm PMMA layer on top. The excitation source is a 385-nm LED with intensity of 120 mW/cm^2. (b) Thermal imaging of the PMMA/crystal/substrate with no illumination. (c) Thermal imaging of the LED illumination spot. (d-f) Thermal imaging of the PMMA/crystal/substrate under illumination for (d) 0 min, (e) 5 min and (f) 10 min.

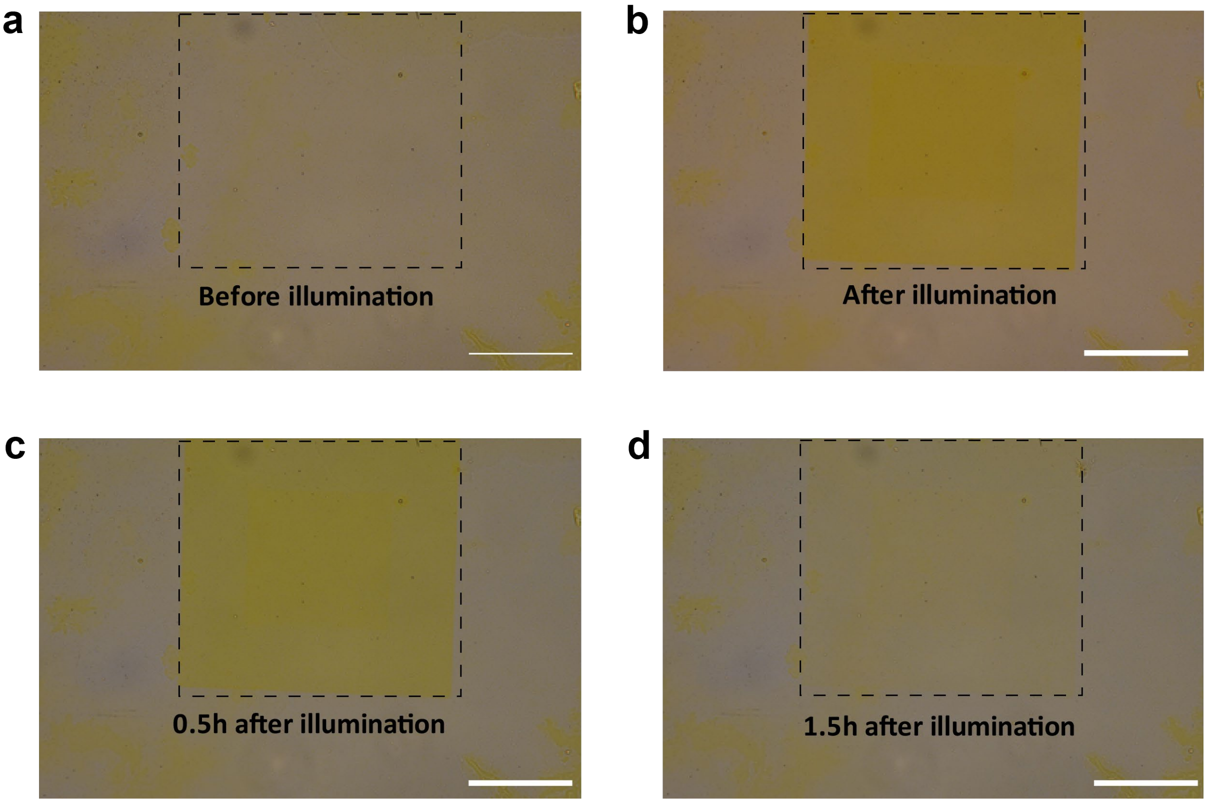

Figure S17. Optical microscopy capturing halide relaxation in the dark for a $BA_2PbBr_2I_2$ single crystal. Optical microscopic images for a $BA_2PbBr_2I_2$ single crystal: (a) Before photoexcitation. (b) After 20 min photoexcitation from a 405 nm LED. (c) 0.5 h in the dark after photoexcitation. (d) 1.5 h in the dark after photoexcitation. The central squared area framed with black dotted line is the photoexcited region. The scale bar is 20 µm. The images are captured in transmission mode. The optical transmittance has been clearly modified right after photoexcitation, indicating halide switching happened. During the afterwards settling in dark, the photoexcited region gradually recovers to close to the original state after 1.5 h.

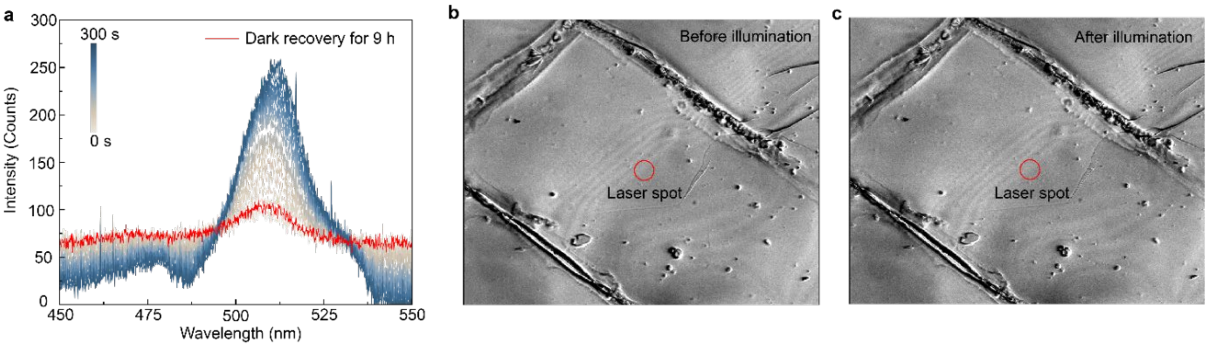

Figure S18. PL dark recovery measurement for a $BA_2PbBr_2I_2$ single crystal. (a) PL spectra capturing halide switching under a 405-nm pulsed laser illumination for 300 s (grey to blue spectra traces) and the PL spectra at the same area measured after 9 h dark treatment (red curve). (b, c) Optical images of the crystal before and after photoexcitation. The red circulated area is the indicates the laser spot.

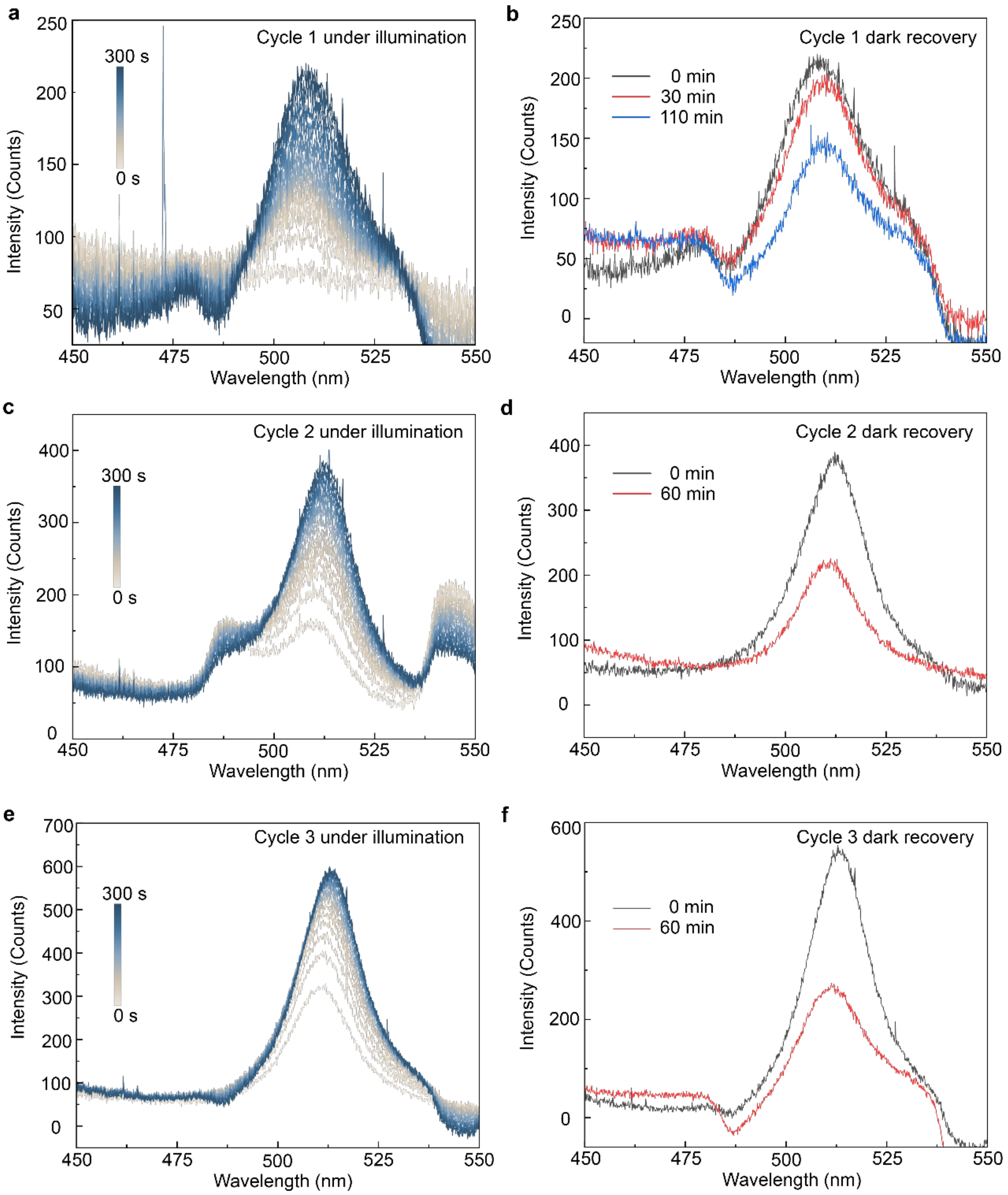

Figure S19. Halide switching and recovery measurement at the same region of interest (ROI) under the same illumination condition. (a, c, e) Time-dependent PL for halide switching: (a) cycle 1, (c) cycle 2 and (e) cycle 3. (b, d, f) Single PL spectra for dark recovery: (b) cycle 1, (d) cycle 2 and (f) cycle 3. Time-dependent PL results for three photoexcitation/dark-recovery cycles on the same laser-focused ROI are shown above. After each cycle of photoexcitation, the sample was rested in the dark until the PL peak reduced to 50±10% of its maximum before processing the next photoexcitation cycle.

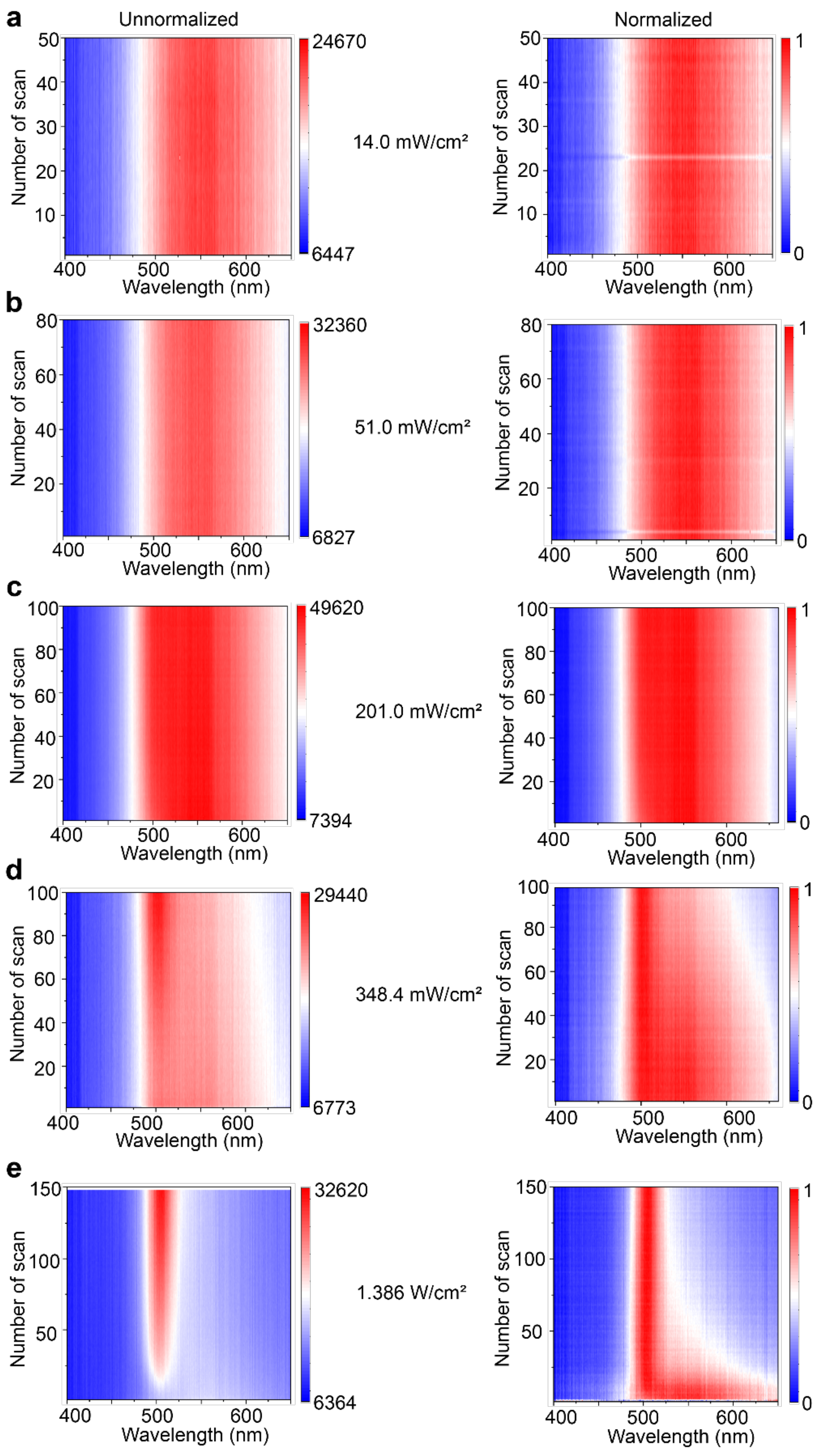

Figure S20. Time dependent PL spectra for BA_2PbBr_3I at different excitation fluences. (a) 14.0 mW/cm^2. (b) 51.0 mW/cm^2. (c) 201.0 W/cm^2. (d) 348.4 mW/cm^2. (e) 1.386 W/cm^2. The spectra on the left column are original spectra and on the right column are normalised spectra. Excitation: a halogen lamp with a 405 nm long pass filter.

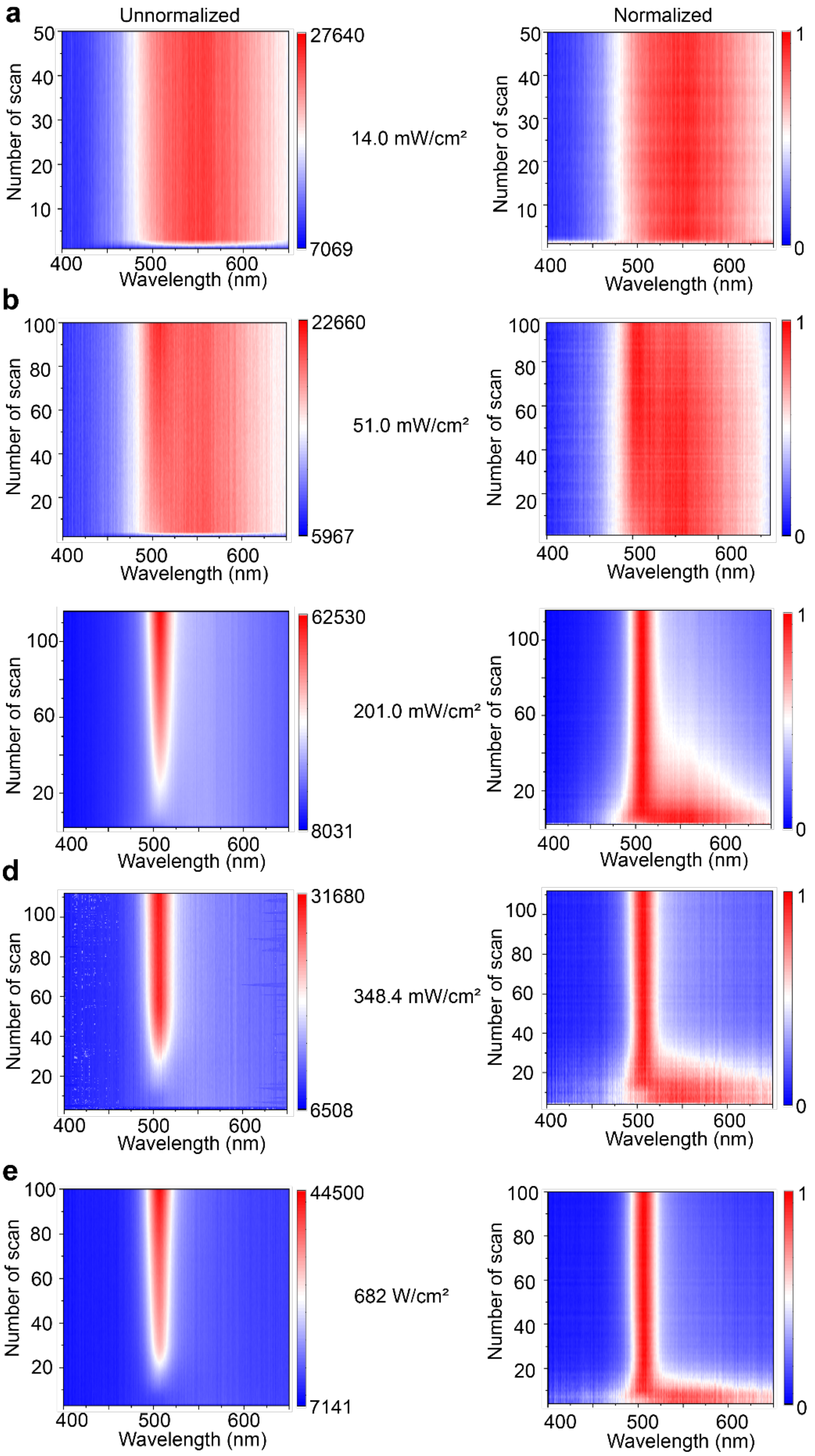

Figure S21. Time dependent PL spectra for $BA_2PbBr_2I_2$ at different excitation fluences. (a) 14.0 mW/cm^2. (b) 51.0 mW/cm^2. (c) 201.0 W/cm^2. (d) 348.4 mW/cm^2. (e) 682 W/cm^2. The spectra on the left column are original spectra and on the right column are normalised spectra. Excitation: a halogen lamp with a 405 nm long pass filter.

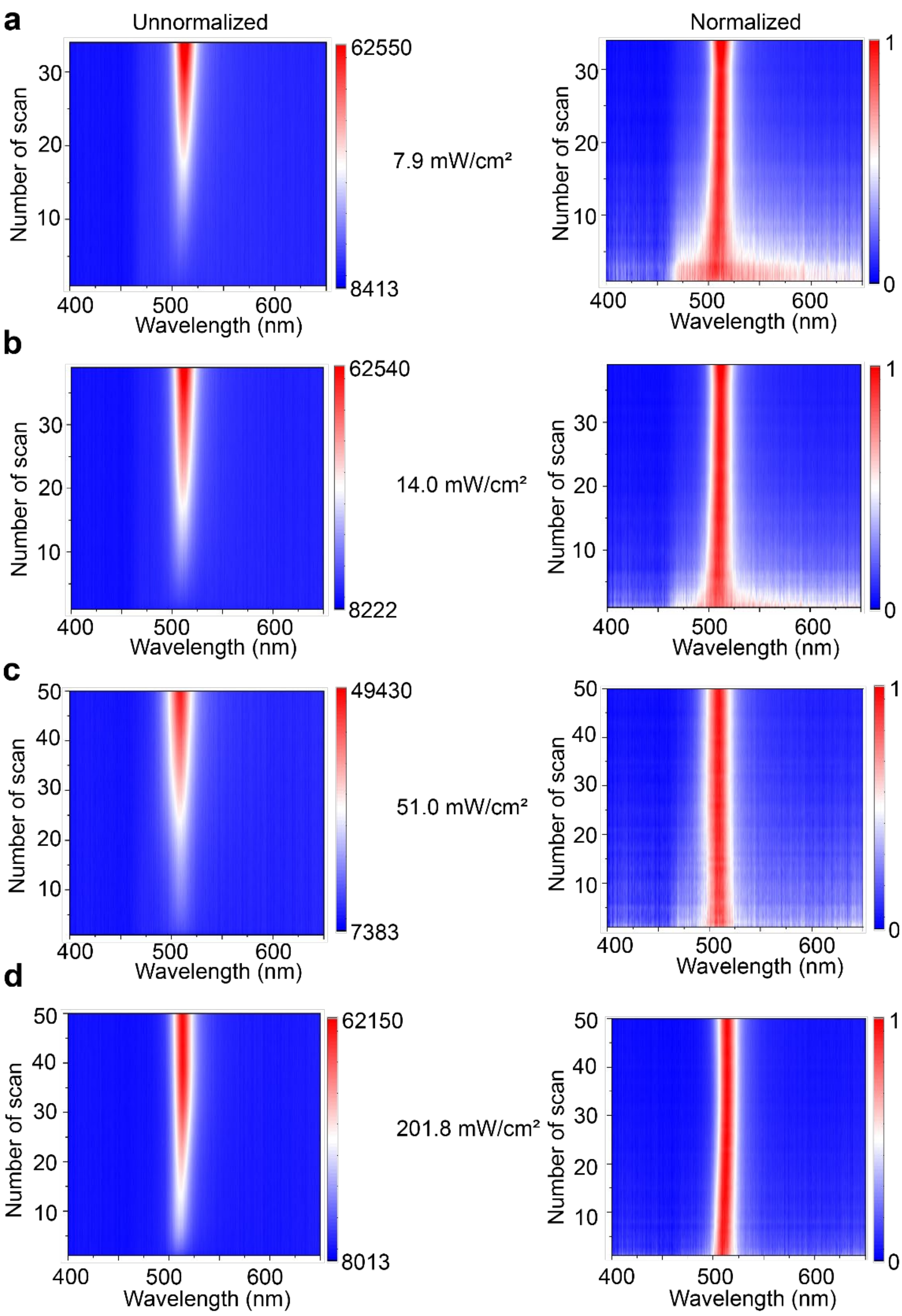

Figure S22. Time dependent PL spectra for BA_2PbBrI_3 at different excitation fluences. (a) 7.9 mW/cm^2. (b) 14.0 mW/cm^2. (c) 51.0 W/cm^2. (d) 201.8 mW/cm^2. The spectra on the left column are original spectra and on the right column are normalised spectra. Excitation: a halogen lamp with a 405 nm long pass filter.

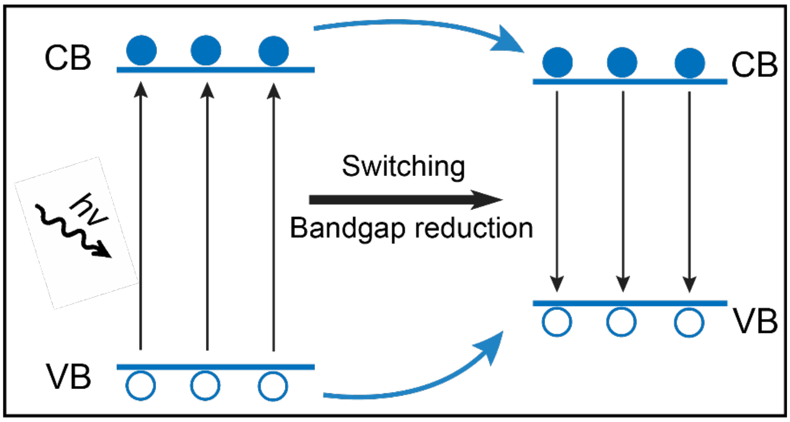

Figure S23. Mechanism illustration of exciton-funnelling from original phase to halide switched phase.

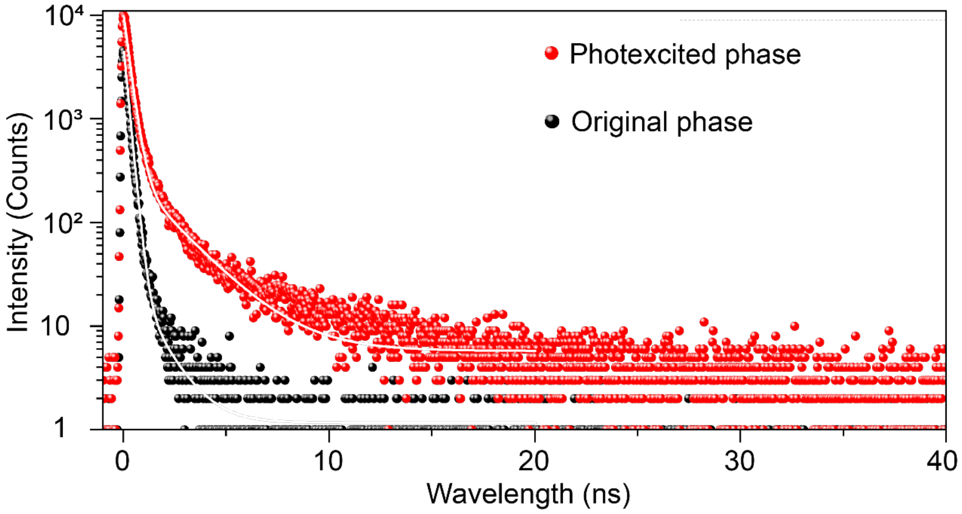

Figure S24. TRPL measurement on a $BA_2PbBr_2I_2$ single crystal for both the original and photoexcited phases. The plots in red and black spheres are TRPL data for the photoexcited phase and original phase, respectively. The white curves are the fit curves. The fitting is performed using a biexponential decay fitting function. We note the biexponential fitting is to mathematically describe the data.

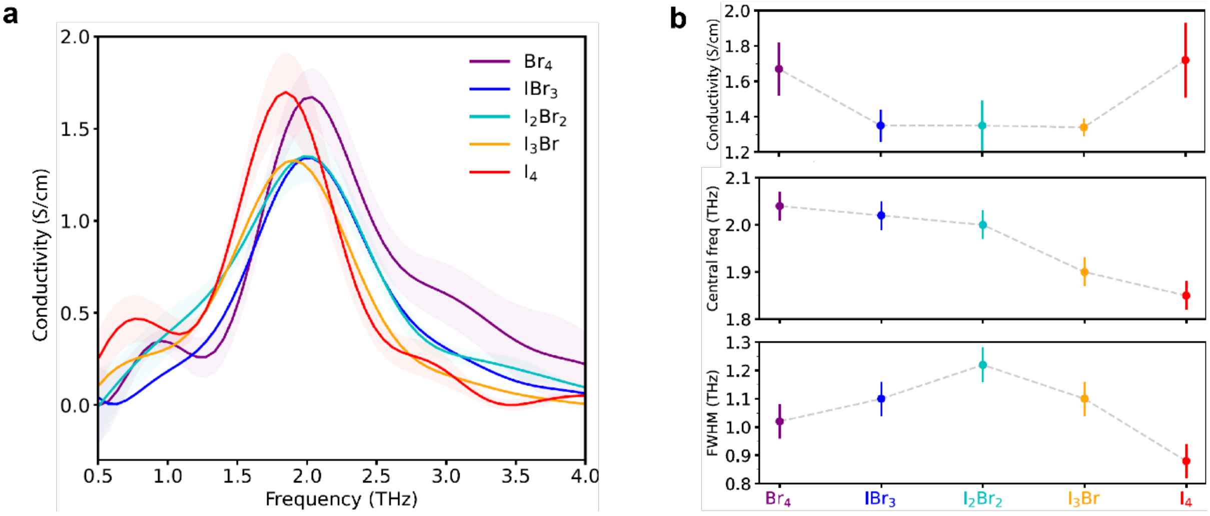

Figure S25. THz conductivity of $BA2PbBr_xI_{4-x}$. (a) Real part of THz conductivity as a function of frequency, for varying halide composition, retrieved via THz time-domain spectroscopy (THz-TDS). See Methods in main text. (b) Conductivity maximum, central frequency and FWHM of ~ 2 THz peak in (a), as a function of halide composition, determined via fitting with a single Lorentzian. A prominent peak is observed in the real part of the THz conductivity, at a frequency increasing with Br content, from 1.8 THz for pure I to 2.15 THz for pure Br. Based on previous THz-TDS studies of 3D perovskites,[6,7] this peak can be assigned to phonon modes arising from the twisting and stretching of the halide octahedra. We observed a reduction of the magnitude and a broadening of this peak as the perovskite composition varied from pure (I or Br) to mixed. This reduction and broadening are likely a result of reduced crystallinity from the less ordered mixed halide structures.

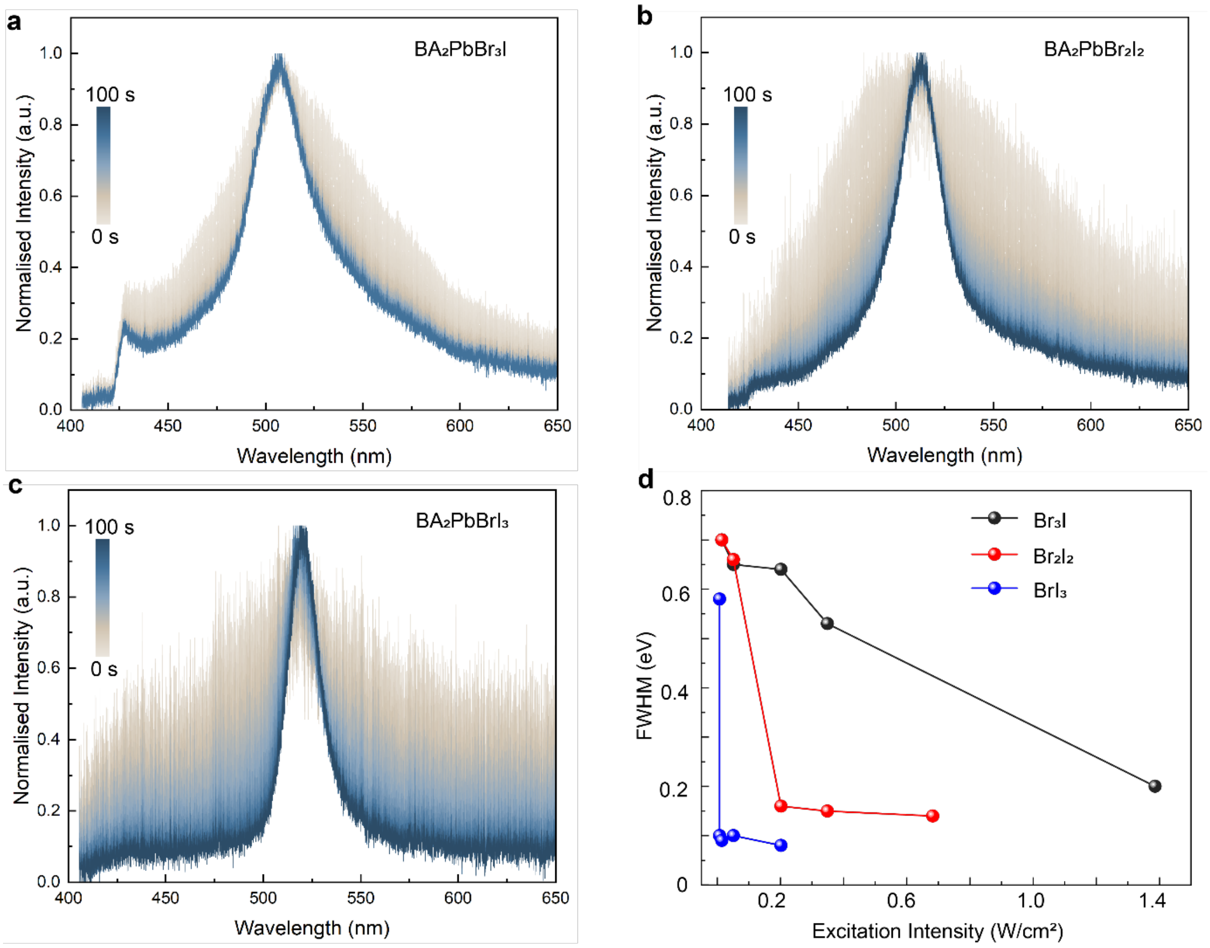

Figure S26. PL FWHM changes at different excitation densities. (a-c) FWHM changes at above threshold excitation densities for different halide ratios. (d) FWHM changes as a function of different excitation fluences for all three halide compositions.

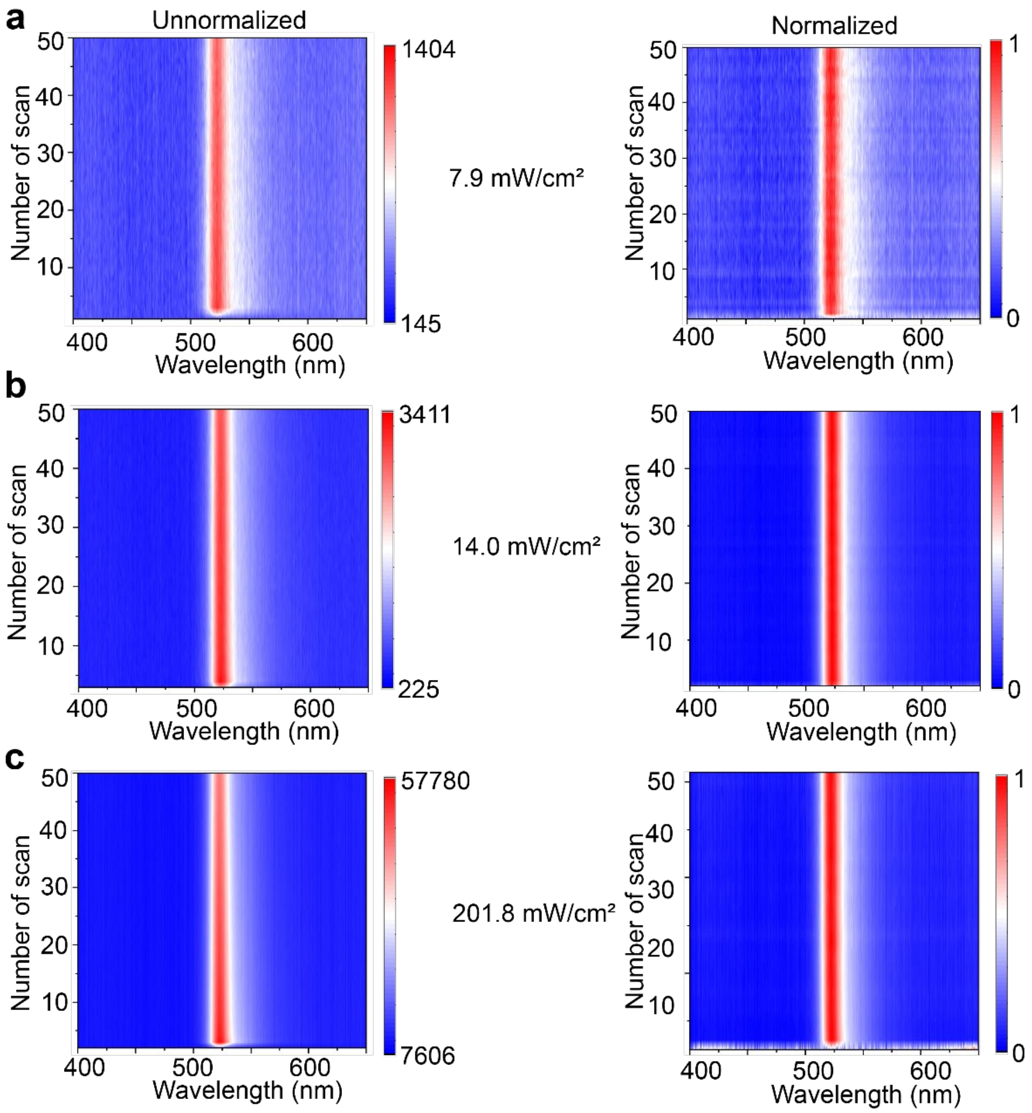

Figure S27. Time dependent PL spectra for BA_2PbI_4 at different excitation fluences. (a) 7.9 mW/cm^2. (b) 14.0 mW/cm^2. (c) 51 W/cm^2. The spectra on the left column are original spectra and on the right column are normalised spectra. Excitation: a halogen lamp with a 405 nm long pass filter.

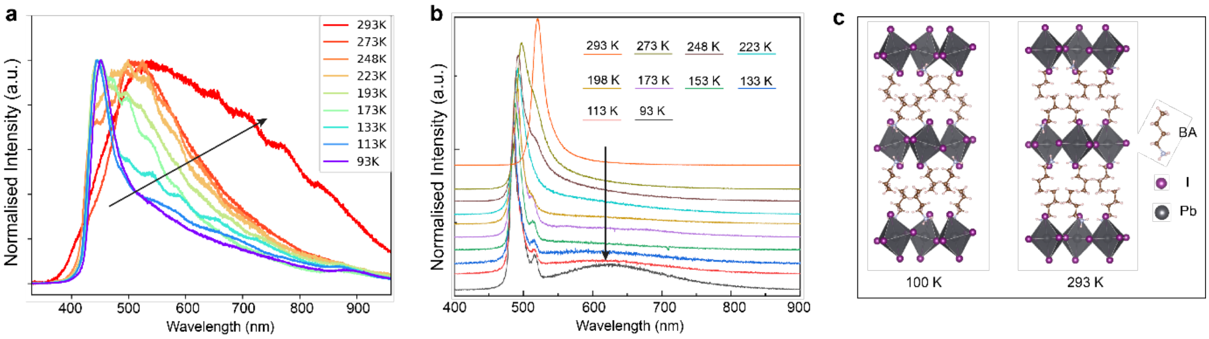

Figure S28. Temperature-dependent CL measurement for $BA_2PbBr_xI_{4-x}$ single crystals. (a) BA_2PbBr_3I. (b) BA_2PbI_4. (c) Diagram of single crystal structures for BA_2PbI_4 measured at 100 K and 293 K. During the measurement, the electron beam was defocused to a larger spot with diameter of around 15 μm to reduce electron beam knock-on damage. In Br_3I, a sharp emission peak was observed at 93 K, which is associated with the free exciton recombination. Upon an increase in temperature, a gradual peak broadening dominates the emission, which is attributed to the self-trapped excitons due to thermally activated exciton-lattice interactions. In contrast in BA_2PbI_4, no peak broadening is observed as temperature increases above 100 K. However, peak broadening is observed as temperature decreases to below 93 K. This suggests that a more distorted structure could induce exciton trapping that contributes to the broadened emission peak. This is consistent with a more distorted lattice structure measured at 100 K compared to a less distorted structure at 293 K.

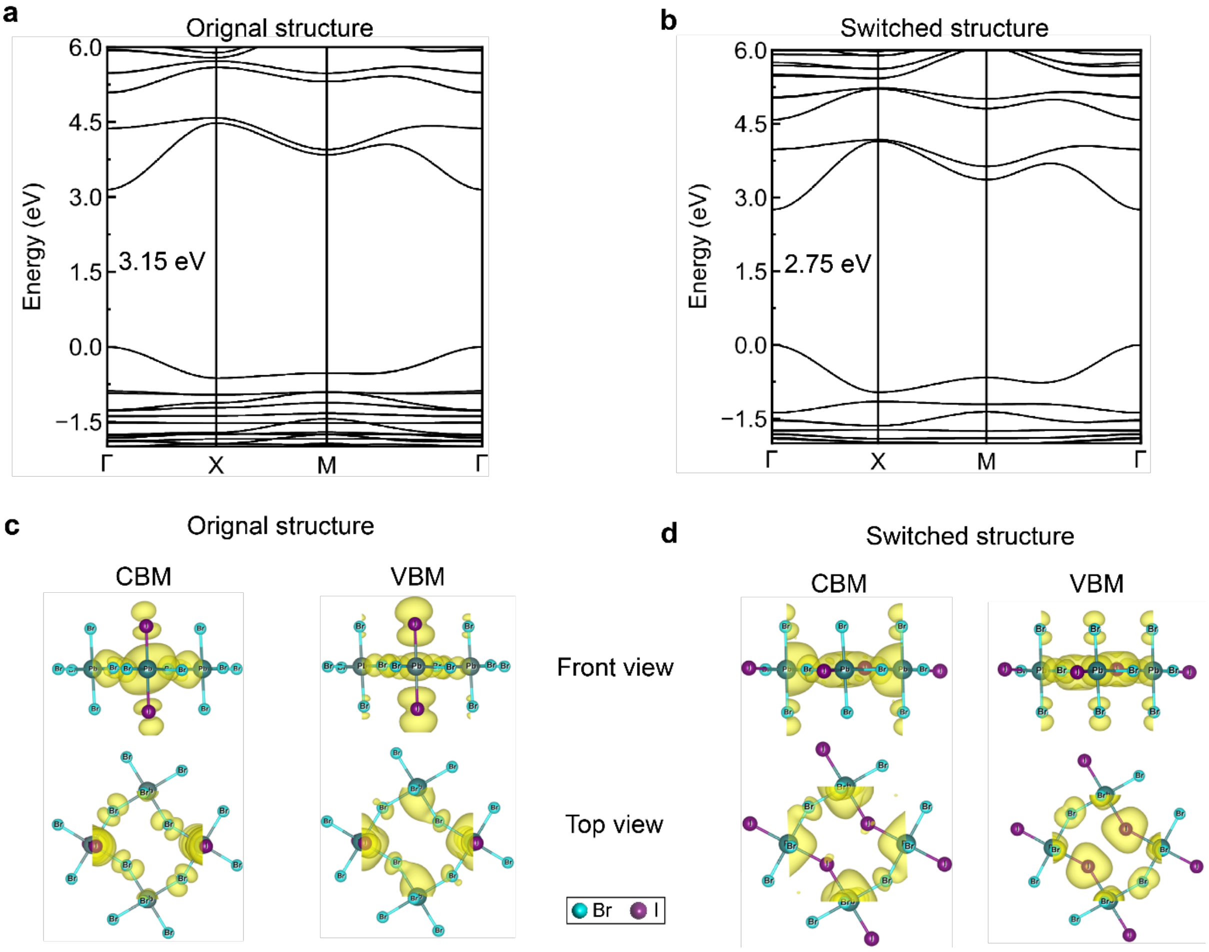

Figure S29. Calculated electronic band structures of BA_2PbBr_3I before and after halide switching. (a-b) Bandgap estimation using GW+SOC method for (a) original structure, and (b) switched structure. (c-d) Corresponding band-decomposed charge density distribution of the conduction band minima (CBM) and valance band maxima (VBM) for (c) original structure and (d) halide switched structure.

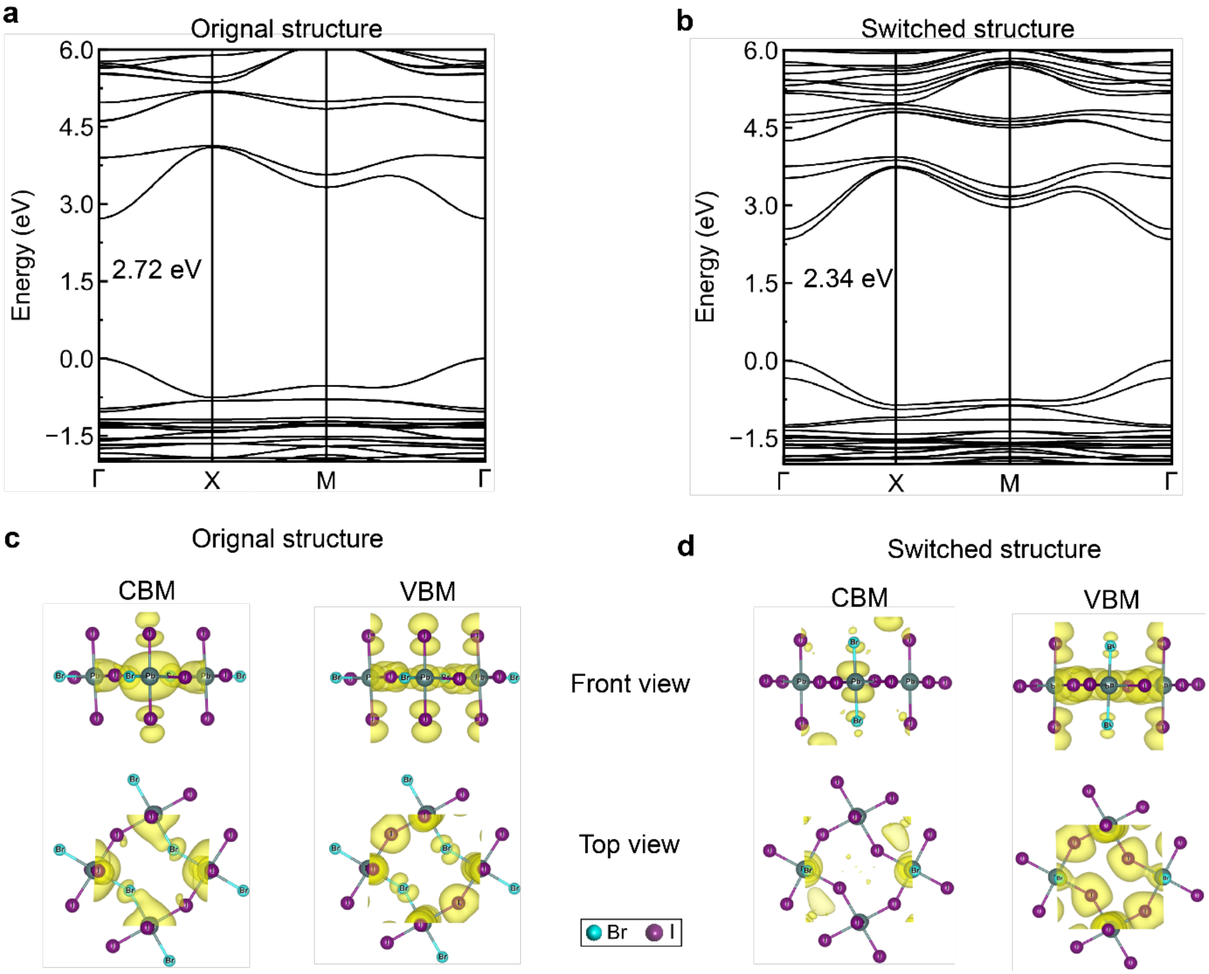

Figure S30. Calculated electronic band structures of BA_2PbBrI_3 before and after halide switching. (a-b) Bandgap estimation using GW+SOC method for (a) original structure, and (b) switched structure. (c-d) Corresponding band-decomposed charge density distribution of the conduction band minima (CBM) and valance band maxima (VBM) for (c) original structure and (d) halide switched structure.

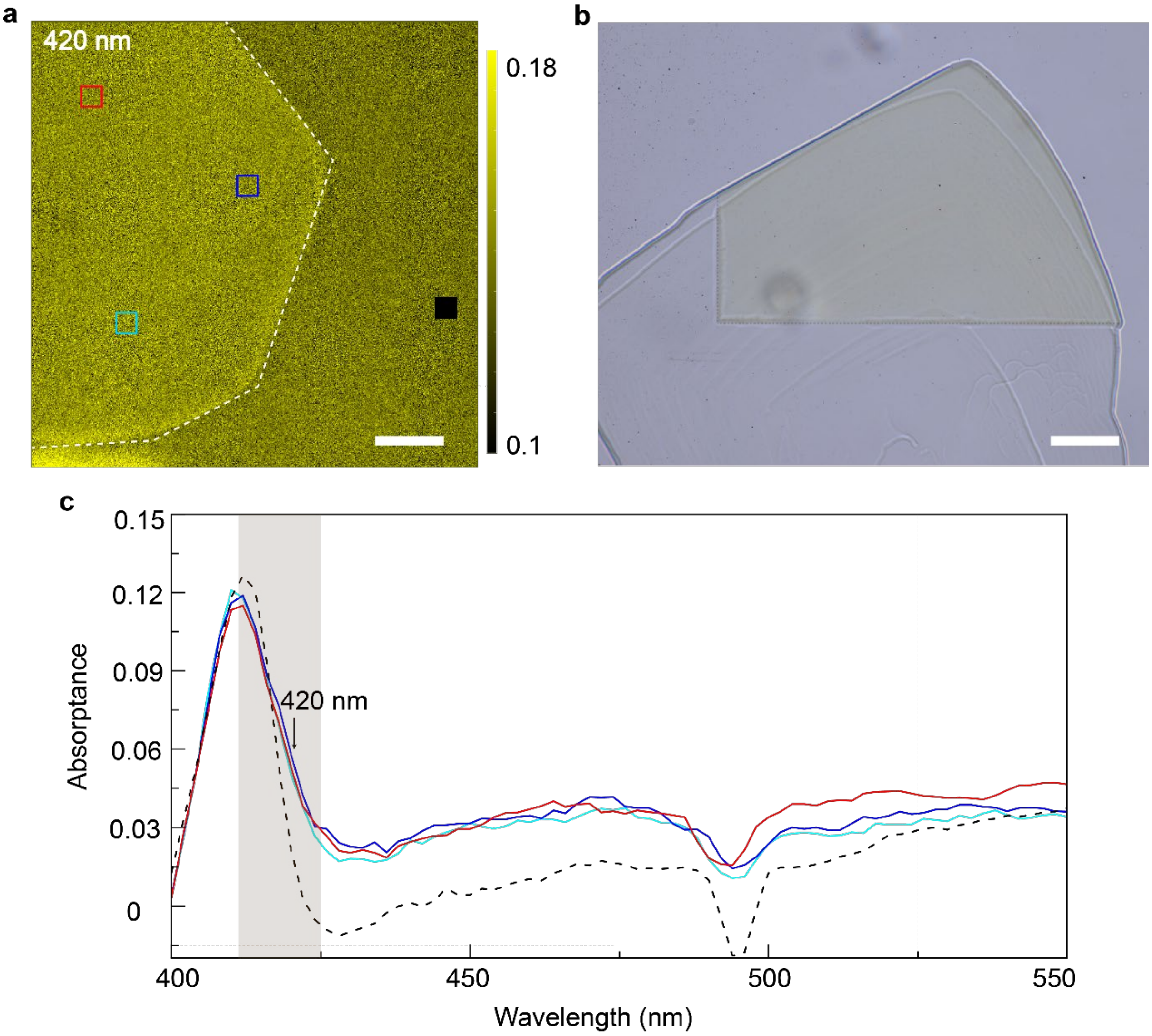

Figure S31. Spatial absorptance measurement for BA_2PbBr_3I single crystals. (a) Spatial absorptance map of a thin BA_2PbBr_3I single crystal. The white dashed line separates the original phase (right side) and photoexcited phase (left side). The map is presented at the central wavelength of 420 nm. (b) Optical microscopic image of a BA_2PbBr_3I single crystal taken in the bright field transmission mode by a halogen lamp. The crystal is optically transparent with close colour contrast to the background due to the large bandgap of BA_2PbBr_3I. The top corner of the crystal with yellowish colour is the region after photoexcitation. A homogeneous ion-switched phase is obtained with no indication of PHS domains. (c) Absorptance spectra for the separate ROIs from the spatial map in (a). The absorptance spectra are measured for three separate points with colour coded boxes at the photoexcited region and a solid black box in the original phase region as shown in (a). In the photoexcited region, the three ROIs show almost the same absorption curves with no homogeneity difference. In addition, there is a slight red shift of the absorption onset compared to the original phase (black dashed curve) indicating a reduction in optical bandgap. The scale bars are 20 μm.

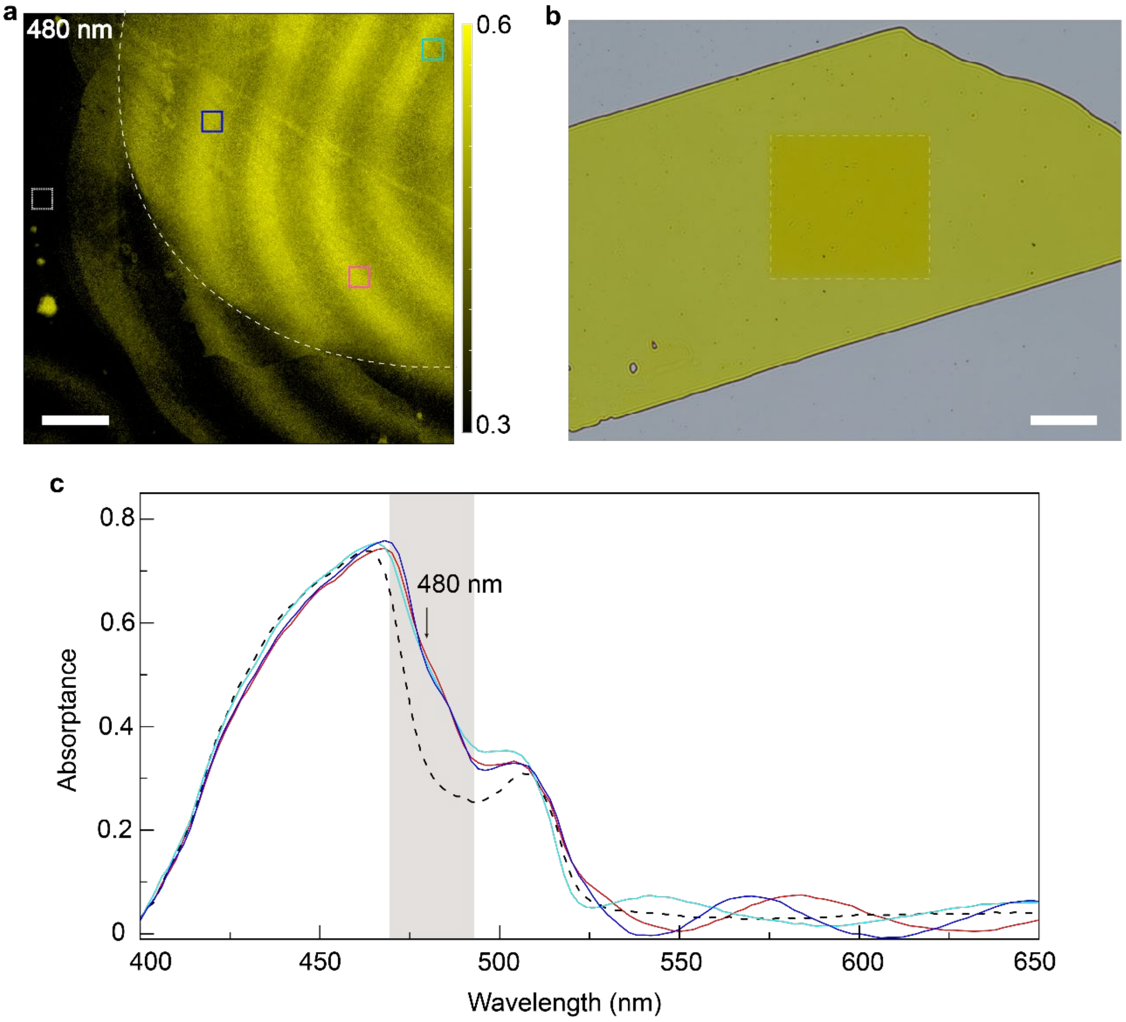

Figure S32. Spatial absorption measurement for BA_2PbBrI_3 single crystals. (a) Spatial absorption map of a thin BA_2PbBr_3I single crystal. The white dashed line separates the original phase (left side) and photoexcited phase (right side). The map is presented at the central wavelength of 480 nm. The fringe patterns in the image are likely due to interference of light reflected from the interfaces between the flat 2D perovskite layers and with the glass substrate. (b) Bright field optical microscopic image of a BA_2PbBrI_3 single crystal in transmission mode. The central squared region in white dashed box presenting slightly darkened orange colour is the region after photoexcitation whereas the rest of the crystal presents light yellowish colour. Similar to Br_3I and Br_2I_2, a homogeneous ion-switched phase instead of PHS domains is obtained. (c) Absorptance spectra for the separate ROIs from the spatial map in (a). The absorptance spectra are measured for three separate points with colour coded boxes at the photoexcited region and a solid black box in the original phase region as shown in (a). In the photoexcited region, the three ROIs show almost same absorption curves with no homogeneity difference. In addition, there is a slight red shift of the absorption onset compared to the original phase (black dashed curve) indicating a reduction in optical bandgap. Note that a secondary absorption peak at between 505 nm to 510 nm are observed in both the original phase and photoexcited phase, which is attributed to interference effect. The scale bars are 20 μm.

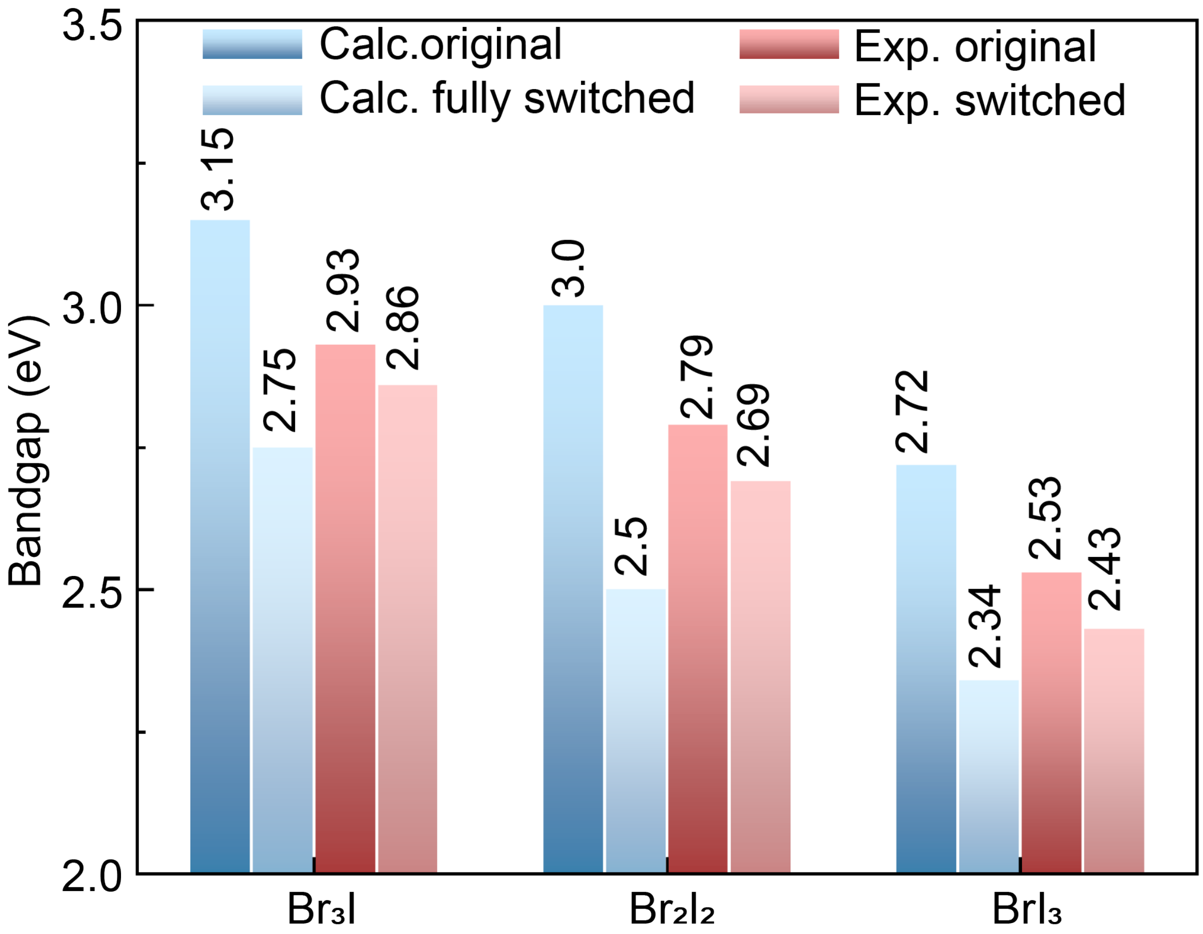

Figure S33. Comparison of DFT-calculated electronic bandgap changes and experimentally observed optical bandgap changes before and after halide switching.

Reference

(1) Smith, M. D.; Jaffe, A.; Dohner, E. R.; Lindenberg, A. M.; Karunadasa, H. I. Structural Origins of Broadband Emission from Layered Pb–Br Hybrid Perovskites. Chemical Science **2017**, *8* (6), 4497-4504.

(2) Dutta, S. K.; Dutta, A.; Das Adhikari, S.; Pradhan, N. Doping Mn^{2+} in Single-Crystalline Layered Perovskite Microcrystals. ACS Energy Letters **2019**, *4* (1), 343-351.

(3) Mao, W.; Hall, C. R.; Bernardi, S.; Cheng, Y.-B.; Widmer-Cooper, A.; Smith, T. A.; Bach, U. Light-Induced Reversal of Ion Segregation in Mixed-Halide Perovskites. *Nat. Mater.* **2021**, *20* (1), 55–61.

(4) Mao, W.; Hall, C. R.; Chesman, A. S. R.; Forsyth, C.; Cheng, Y.-B.; Duffy, N. W.; Smith, T. A.; Bach, U. Visualizing Phase Segregation in Mixed-Halide Perovskite Single Crystals. *Angew. Chem., Int. Ed.* **2019**, *58* (9), 2893–2898.

(5) Motti, S. G.; Patel, J. B.; Oliver, R. D. J.; Snaith, H. J.; Johnston, M. B.; Herz, L. M. Phase Segregation in Mixed-Halide Perovskites Affects Charge-Carrier Dynamics while Preserving Mobility. *Nat. Commun.* **2021**, *12* (1), 6955.

(6) Hoke, E. T.; Slotcavage, D. J.; Dohner, E. R.; Bowring, A. R.; Karunadasa, H. I.; McGehee, M. D. Reversible Photo-Induced Trap Formation in Mixed-Halide Hybrid Perovskites for Photovoltaics. *Chem. Sci.* **2015**, *6* (1), 613–617.

(7) Bischak, C. G.; Hetherington, C. L.; Wu, H.; Aloni, S.; Ogletree, D. F.; Limmer, D. T.; Ginsberg, N. S. Origin of Reversible Photoinduced Phase Separation in Hybrid Perovskites. *Nano Lett.* **2017**, *17* (2), 1028–1033.

(8) Leguy, A. M. A.; Goñi, A. R.; Frost, J. M.; Skelton, J.; Brivio, F.; Rodríguez-Martínez, X.; Weber, O. J.; Pallipurath, A.; Alonso, M. I.; Campoy-Quiles, M.; et al. Dynamic Disorder, Phonon Lifetimes, and the Assignment of Modes to the Vibrational Spectra of Methylammonium Lead Halide Perovskites. *Phys. Chem. Chem. Phys.* **2016**, *18* (39), 27051–27066.

(9) Brivio, F.; Frost, J. M.; Skelton, J. M.; Jackson, A. J.; Weber, O. J.; Weller, M. T.; Goñi, A. R.; Leguy, A. M. A.; Barnes, P. R. F.; Walsh, A. Lattice Dynamics and Vibrational Spectra of the Orthorhombic, Tetragonal, and Cubic Phases of Methylammonium Lead Iodide. *Phys. Rev. B* **2015**, *92* (14), 144308.
